# CRISPR/Cas9-Induced *fad2* and *rod1* Mutations Stacked With *fae1* Confer High Oleic Acid Seed Oil in Pennycress (*Thlaspi arvense* L.)

**DOI:** 10.3389/fpls.2021.652319

**Published:** 2021-04-22

**Authors:** Brice A. Jarvis, Trevor B. Romsdahl, Michaela G. McGinn, Tara J. Nazarenus, Edgar B. Cahoon, Kent D. Chapman, John C. Sedbrook

**Affiliations:** ^1^School of Biological Sciences, Illinois State University, Normal, IL, United States; ^2^BioDiscovery Institute and Department of Biological Sciences, University of North Texas, Denton, TX, United States; ^3^Department of Biochemistry and Center for Plant Science Innovation, University of Nebraska-Lincoln, Lincoln, NE, United States

**Keywords:** CRISPR, MALDI-MSI, oilseed, oleic acid, pennycress, polyunsaturated fatty acid, *Thlaspi arvense*, triacylglycerol

## Abstract

Pennycress (*Thlaspi arvense* L.) is being domesticated as an oilseed cash cover crop to be grown in the off-season throughout temperate regions of the world. With its diploid genome and ease of directed mutagenesis using molecular approaches, pennycress seed oil composition can be rapidly tailored for a plethora of food, feed, oleochemical and fuel uses. Here, we utilized Clustered Regularly Interspaced Short Palindromic Repeats (CRISPR)/Cas9 technology to produce knockout mutations in the *FATTY ACID DESATURASE2* (*FAD2*) and *REDUCED OLEATE DESATURATION1* (*ROD1*) genes to increase oleic acid content. High oleic acid (18:1) oil is valued for its oxidative stability that is superior to the polyunsaturated fatty acids (PUFAs) linoleic (18:2) and linolenic (18:3), and better cold flow properties than the very long chain fatty acid (VLCFA) erucic (22:1). When combined with a *FATTY ACID ELONGATION1* (*fae1*) knockout mutation, *fad2 fae1* and *rod1 fae1* double mutants produced ∼90% and ∼60% oleic acid in seed oil, respectively, with PUFAs in *fad2 fae1* as well as *fad2* single mutants reduced to less than 5%. MALDI-MS spatial imaging analyses of phosphatidylcholine (PC) and triacylglycerol (TAG) molecular species in wild-type pennycress embryo sections from mature seeds revealed that erucic acid is highly enriched in cotyledons which serve as storage organs, suggestive of a role in providing energy for the germinating seedling. In contrast, PUFA-containing TAGs are enriched in the embryonic axis, which may be utilized for cellular membrane expansion during seed germination and seedling emergence. Under standard growth chamber conditions, *rod1 fae1* plants grew like wild type whereas *fad2* single and *fad2 fae1* double mutant plants exhibited delayed growth and overall reduced heights and seed yields, suggesting that reducing PUFAs below a threshold in pennycress had negative physiological effects. Taken together, our results suggest that combinatorial knockout of *ROD1* and *FAE1* may be a viable route to commercially increase oleic acid content in pennycress seed oil whereas mutations in *FAD2* will likely require at least partial function to avoid fitness trade-offs.

## Introduction

There is overwhelming evidence that the use of fossil fuels is driving climate change, the mitigation of which may benefit from replacement with increased production of biofuels derived from oilseed crops grown sustainably (Vollmann and Rajcan, 2009; Fan et al., 2013; IPCC, 2013; Hill et al., 2016; Drake et al., 2017; Masson-Delmotte et al., 2018). Demands on oilseed production for food and other uses are also intensifying due to population growth and improved standards of living. Two well-established oilseed crops are soybean (*Glycine max* L.) and canola (*Brassica napus*), producing over 400 million metric tons of seed, annually, rich in both oil and protein (USDA, United States Department of Agriculture Foreign Agriculture Services, 2013). The oils from these crops can be used in a plethora of products including foodstuffs, feed, cosmetics, and industrial lubricants along with biodiesel and biojet fuels (Dyer et al., 2008; Volkova et al., 2016). However, it is important to balance needs for human nutrition with those of bio-based products. Taken together, there is impetus to develop new oilseed crops that can be grown with relatively low greenhouse gas emissions and without causing detrimental land use changes (Fulton et al., 2015).

Pennycress (*Thlaspi arvense* L.) is an emerging oilseed crop and member of the Brassicaceae family (mustard family), native to Eurasia and naturalized throughout temperate regions of the world (Mc Intyre and Best, 1978; Sharma et al., 2007). Pennycress can be grown as a winter annual cover crop on otherwise fallow farmland, providing ecosystems benefits and avoiding displacement of established food crops (Isbell, 2008; Phippen and Phippen, 2012; Fan et al., 2013). Pennycress is self-pollinating with a diploid genome that shares ∼86% nucleotide sequence identity with the genome of the well-studied model organism *Arabidopsis thaliana* (Arabidopsis) (Dorn et al., 2013, 2015). Like Arabidopsis, pennycress is well-suited for laboratory studies, growing well side by side with Arabidopsis in growth chambers and greenhouses and being readily genetically transformable using an *Agrobacterium tumefaciens*-mediated floral dip method, albeit requiring vacuum application during the floral dip (McGinn et al., 2019). Ease of pennycress mutagenesis using ethyl methanesulfonate (EMS) as well as Clustered Regularly Interspaced Short Palindromic Repeats (CRISPR)/Cas9 gene editing, along with its ease of growth and study in farm field settings, makes pennycress an attractive multifunctional model system for simultaneous basic and applied research (Sedbrook et al., 2014; Chopra et al., 2018, 2020; Sedbrook and Durrett, 2020; Tsogtbaatar et al., 2020).

Pennycress seeds naturally accumulate high amounts of oil (∼35%) and protein (∼20%), similar to other established and emerging Brassicaceae oilseed crops such as canola, carinata (*Brassica carinata*), and camelina (*Camelina sativa*) (Moser et al., 2009; Kang et al., 2011; Selling et al., 2013; Hossain et al., 2019; Kumar et al., 2020). Pennycress can yield up to 840 liters of oil per hectare, with a potential of United States Midwest Corn Belt-grown pennycress to replace ∼5% of petroleum sources currently used in the United States (Moser et al., 2009; Boateng et al., 2010; Fan et al., 2013; Mousavi-Avval and Shah, 2020). In addition to possible uses for biofuels, food, and feed production, the seed oil-derived fatty acids (FAs) can be processed to produce wax esters which are used in cosmetics, surfactants, lubricants, and plastics (Volkova et al., 2016). A main objective of research on pennycress is altering its seed lipid profile to create value-added feedstocks better suited for specific applications. For example, pennycress seed TAGs naturally contain high amounts of very long chain fatty acids (VLCFAs) mostly in the form of erucic acid (22:1; ∼35%), which could be genetically enhanced further through breeding and engineering (Li et al., 2012; Guan et al., 2014; Altendorf et al., 2019; Claver et al., 2020). Erucic acid has multiple industrial uses and is suitable for hydrocracking to produce dodecane (C11) and other medium and long chain fatty acids (MCFAs, LCFAs) for aviation fuel and biodiesel (Bezergianni and Kalogianni, 2009; Sotelo-Boyás et al., 2012; Isbell et al., 2015). Pennycress can also be engineered to produce seed TAGs and acetyl-TAGs containing MCFAs suitable for industrial, biojet fuel and improved biodiesel applications (Esfahanian et al., 2021). It has also been shown in different plant species that specific mutations and mutation combinations can substantially decrease VLCFAs and polyunsaturated fatty acids (PUFAs, e.g., 18:2 linoleic acid and 18:3 linolenic acid) thereby increasing 18:1 oleic acid content in seed oil, which is a desirable biodiesel component (Sivaraman et al., 2004; Kang et al., 2011; McGinn et al., 2019; Chopra et al., 2020; Neumann et al., 2021). Oleic acid has increased oxidative stability compared to linoleic acid and linolenic acid thereby improving fuel stability as well as improving the oil’s suitability for high-temperature food applications such as frying (Frega et al., 1999; Serrano et al., 2014; Lanjekar and Deshmukh, 2016). Oleic acid also has better cold-flow properties than VLCFAs, making it more suitable as a component of biodiesel (Stournas et al., 1995; Verma et al., 2016; Sia et al., 2020). From a nutritional motivation, erucic acid levels are limited in food and feed due to potential toxicity when eaten in large amounts (Millar and Kunst, 1997), so reductions in VCLFA amounts in pennycress could be beneficial from this standpoint.

**FIGURE 1:**
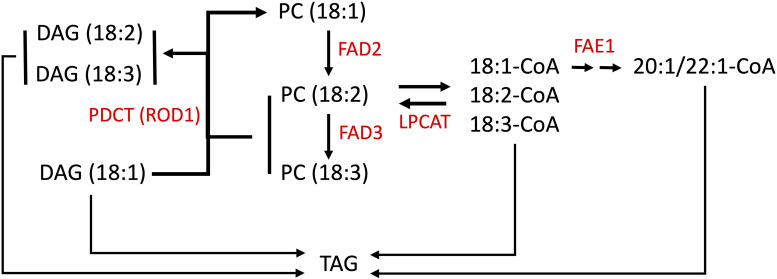
Simplified scheme for fatty acid modification in Brassicas. Fatty acid (FA) elongation of 18:1-CoA is carried out by a protein complex containing the FA elongase, FAE1. Lysophosphatidylcholine acyltransferase (LPCAT) shuttles acyl groups between PC and the acyl-CoA pool. The polyunsaturated FA pathway involves desaturation of 18:1 (esterified to PC) by the FA desaturases, FAD2 and FAD3. 18:1-CoA and acyl-PC are the substrates for FAE1 and the FA desaturases, respectively. PDCT (phosphatidylcholine (PC):diacylglycerol (DAG) cholinephosphotransferase) is also named REDUCED OLEATE DESATURATION (ROD1) that modulates polyunsaturated FA (PUFA) content by interconverting PC and DAG, transferring 18:1 into PC for desaturation, and 18:2 and 18:3 into the triacylglycerol (TAG) biosynthetic pathway thereby increasing PUFA content in TAG (Lu et al., 2009). Choline phosphotransferase (CPT) may also mediate the interconversion of DAG and PC but is not shown here for simplicity.

Pennycress seeds store lipids as TAGs within embryonic tissues, which serve as an energy source for the seed embryo upon germination (Goffman et al., 2005; Tsogtbaatar et al., 2020). TAGs are composed of a glycerol backbone with three ester-linked fatty acid carbon chains of different lengths and levels of unsaturation (Chapman and Ohlrogge, 2012). Oleic acid linked to CoA is converted by the enzyme FAE1 (FATTY ACID ELONGATION1) to eicosenoic acid then erucic acid (Figure 1). Oleic acid esterified to PC is also converted to linoleic acid by the enzyme FATTY ACID DESATURASE2 (FAD2), which is further reduced to linolenic acid by FATTY ACID DESATURASE3 (FAD3) (Figure 1; Browse and Somerville, 1991; Browse et al., 1993; Okuley et al., 1994). Linoleic and linolenic acids are omega-6 and omega-3 PUFAs, respectively, shown to have dietary “heart-health” benefits when balanced in the diet (Simopoulos, 2011; de Lorgeril and Salen, 2012; Ruiz-Núñez et al., 2013). Claver et al. (2017, 2020) performed enzymatic and functional analyses on the pennycress FAE1 enzyme, in the process finding that pennycress embryos incorporate erucic acid into TAG very early during seed maturation, concomitant with decreased desaturase activity, and that erucic acid is preferentially incorporated into TAG via DGAT1. Mutations in *FAE1* can block seed oil erucic and eicosenoic acids production resulting in increased oleic, linoleic, and linolenic acids content, whereas mutations in *FAD2* genes have been found in different species to reduce seed oil PUFAs content and increase oleic acid content (Demorest et al., 2016; Li et al., 2016; Jiang et al., 2017; Lakhssassi et al., 2017).

*ROD1* (*REDUCED OLEATE DESATURASE1*) is another gene involved with modulating unsaturated fatty acid levels (reviewed in Bates, 2016). The *ROD1* gene encodes a phosphatidylcholine:diacylglycerol cholinephosphotransferase (PDCT) which interconverts diacylglycerol (DAG) and phosphatidylcholine (PC). ROD1 transfers a phosphocholine from a PC molecule to the *sn*-3 position of a DAG molecule, and acts as a gatekeeper enzyme by directing 18:1 molecules for further desaturation on PCs (Figure 1). It also provides a mechanism for returning the products (18:2 and 18:3) back into the DAG pool for membrane and storage lipid synthesis (Lu et al., 2009; Bates and Browse, 2012; Bai et al., 2020). In addition to the action of PDCT on PC, an acyl editing cycle catalyzed by lyso phosphatidylcholine acyltransferase (LPCAT) shuttles fatty acids between PC and the acyl-CoA pools, and this pathway plays an important role in providing unsaturated acyl-CoAs for incorporation into TAGs (Figure 1). Hence there are multiple steps which may influence the fatty acid composition of seed oils. Researchers discovered in Arabidopsis that loss-of-function mutations in the *rod1* gene reduced the amounts of PUFAs produced. Further, combining either a *rod1* or *fad2* mutation with an *fae1* mutation can produce even more dramatic changes to the seed lipid composition, greatly increasing the amounts of oleic acid (Nguyen et al., 2013; Chopra et al., 2020; Neumann et al., 2021).

In this study, our objective was to provide evidence that pennycress seed genotypes with elevated oleic acid content could be produced by the targeted knockout of the putative pennycress *FAD2* and *ROD1* genes using CRISPR gene editing. Further, we combined each single mutation with a CRISPR/Cas9-edited *fae1* knockout mutation to block both desaturation and elongation of oleic acid (Figure 1). Homozygous *fad2* and *rod1* mutants were generated and further gene combinations created through crossing with the previously generated *fae1-3* mutant (McGinn et al., 2019), creating *fad2 fae1* and *rod1 fae1* double knockout mutants. Growth phenotypes and overall fitness of the mutants were characterized, documenting traits such as germination rates, flowering times, and seed yields. Additionally, using GC-FID (Gas Chromatography-Flame Ionization Detection), ESI-MS (electrospray ionization-mass spectrometry), and MALDI-MSI (matrix assisted desorption/ionization-mass spectrometry imaging), we quantified the changes in the different FA species and determined the relative amounts and distributions of PC and TAG molecular species (Sturtevant et al., 2016, 2018; Woodfield et al., 2017) in pennycress wild-type and mutant embryos, which indicated different impacts to overall lipid metabolic processes in each of the mutant genotypes. Our data show that the oleic acid content in pennycress seed TAGs can be substantially increased by combining a *fae1* knockout mutation with either a *fad2* or *rod1* knockout mutation, attaining 90% oleic acid in *fad2 fae1* double mutant seed TAGs. However, *fad2* knockout mutations slowed plant growth considerably, likely due to reducing PUFAs below physiologically important levels.

## Results

### CRISPR/SaCas9-Induced Mutations in the Putative *TaFAD2* Gene Greatly Reduce Linoleic and Linolenic Acid Content in Pennycress Seed TAGs

**FIGURE 2:**
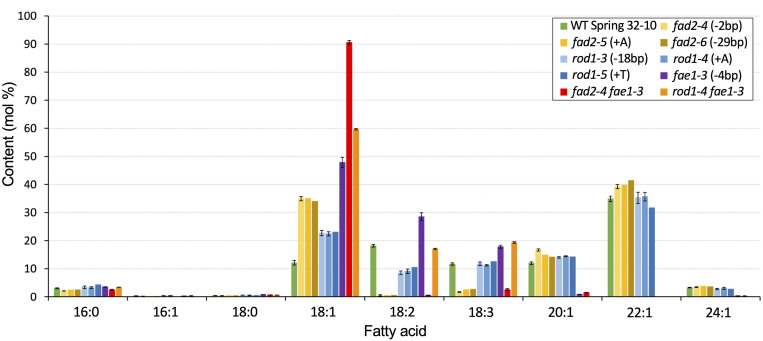
Fatty acid composition of seed TAGs for the different lipid mutants versus WT. Graph shows mean weight percents of the different lipid species. Error bars represent standard deviations of the means where *n* = 2 or 3 biological reps and 3 technical reps. Those without error bars are single samples. See Supplementary Table 1 for values and statistical differences.

The seeds of *Thlaspi arvense* (pennycress) accumulate triacylglycerols (TAGs) containing about 12% oleic acid (18:1), 18% linoleic acid (18:2), and 12% linolenic acid (18:3) (Figure 2 and Supplementary Table 1). The presence of high levels of linoleic and linolenic acids suggest that pennycress has active *FAD2* and *FAD3* fatty acid desaturase genes. Since monounsaturated oleic acid is preferred over the PUFAs for many commercial applications including biodiesel production owing to its superior oxidative stability, we set out to identify the pennycress *TaFAD2* coding sequences and to determine how CRISPR/Cas9 knock out of its function would affect pennycress seed TAG fatty acid composition along with plant and seed health. Using *Arabidopsis thaliana FAD2* (*AtFAD2*; AT3G12120.1) coding sequences as the query, we searched the *T. arvense* genome sequences database using a pennycress-specific Basic Local Alignment Search Tool (BLAST) program available at http://pennycress.umn.edu. We also used *AtFAD2* coding sequences and National Center for Biotechnology Information (NCBI) nucleotide BLAST to search the *T. arvense* transcriptome shotgun assembly database (TSA; Dorn et al., 2013, 2015). Together, these searches identified the putative *TaFAD2* gene and the TSA sequence GAKE01001774.1^1^. Alignment of the *TaFAD2* genomic sequence with the GAKE01001774.1 sequence showed 100% identity and no introns and an open reading frame (ORF) 1,152 nucleotides in length. Sequence alignment also showed that the *TaFAD2* ORF shares 88.8% nucleotide sequence identity with the 1,152 nucleotides *AtFAD2* ORF (AT3G12120.1) (Supplementary Figure 1).

To target knockout of the *TaFAD2* gene, we created a CRISPR/SaCas9 binary vector construct named TaFAD2-CRISPR-Cas9_Hyg, utilizing a vector set containing *Staphylococcus aureus* Cas9 (*Sa*Cas9) that was adapted for use in Arabidopsis by Steinert et al. (2015). The TaFAD2-CRISPR-Cas9_Hyg vector was designed to introduce edits ∼263 nucleotides downstream of the *TaFAD2* translational start site (Supplementary Figure 1). The TaFAD2-CRISPR-Cas9_Hyg vector was introduced into pennycress plants using the *Agrobacterium*-mediated vacuum infiltration method developed by McGinn et al. (2019). Hygromycin selection of the progeny from the *Agrobacterium*-dipped plants identified eight independent T_1_-generation transformants. To screen for Cas9 editing activity, we performed T7 endonuclease I digestion (Pyott et al., 2016) of *FAD2* PCR products amplified from leaf genomic DNA purified from these plants. T7 endonuclease I cuts double-stranded DNA at sites where there is DNA base-pair mismatch, in this case originating from CRISPR/SaCas9-induced mutations. No mismatches were detected in the analyzed T_1_-generation plant tissues. Therefore, we planted out at least 16 T_2_ progeny from three of the T_1_ plants, speculating that SaCas9-induced edits might be detected with the T7 endonuclease method in the T_2_ generation. This approach identified progeny from one of the three T_1_ plants as having putative mutations at the *FAD2* gene target site (Supplementary Figure 2A). Two T_2_ plants were chosen for further analyses based on DNA sequence analysis that showed one was heterozygous for a 2 base-pair (bp) deletion and the other heterozygous for an adenine (A) nucleotide insertion. Both mutations were located as expected at the CRISPR-targeted site within the *FAD2* ORF (Supplementary Figures 1, 2B). These mutations segregated as recessive, with the seeds of homozygous mutants exhibiting altered fatty acid profiles (described below). Moreover, during these analyses, a new *fad2* mutation (29 bp deletion; Supplementary Figure 2B) was detected in one of the plants whose parent had been heterozygous for the 2 bp deletion. The 29 bp deletion likely was introduced in the wild-type *FAD2* allele by continued activity of the CRISPR/SaCas9 machinery. Lines homozygous and stable for each of these three mutations, having the CRISPR/Cas9 construct segregated away, were subsequently isolated and named *fad2-4* (2 bp deletion), *fad2-5* (single A insertion), and *fad2-6* (29 bp deletion) (Supplementary Figure 2B). All three mutations result in frameshifts in the *FAD2* open reading frame and therefore likely confer total loss of *FAD2* function (knockout mutants) by preventing translation of a full-length FAD2 polypeptide (Supplementary Figures 2C–F), resulting in production of truncated fad2 protein and/or causing *fad2* transcript degradation due to nonsense mediated mRNA decay (Conti and Izaurralde, 2005).

Note that pennycress *fad2-1*, *fad2-2*, and *fad2-3* mutant alleles, which are not part of this study, were identified in an ethyl methanesulfonate (EMS) mutant population by screening for altered seed lipid fingerprints using near-infrared spectroscopic (NIRS) analysis. Little has been published on those EMS alleles (Chopra et al., 2019). Also note that all of the mutant lines in this study had the CRISPR/CAS9 constructs segregated away before in-depth genotypic and phenotypic analyses were performed. Moreover, the chosen CRISPR/Cas9 protospacer sequences had less than 70% sequence identity with any other pennycress sequences (based on BLAST searches of the pennycress genome), in order to avoid off-target mutations. In addition, since the *fad2* mutants had strong growth phenotypes in addition to the expected seed lipid compositional changes, we PCR amplified and sequenced the potential chromosomal off-target sites sharing at least 50% sequence identity with the *FAD2* protospacer and, as expected, found no mutations.

Seeds from homozygous *fad2-4*, *fad2-5*, and *fad2-6* plants were analyzed for TAG fatty acid composition, revealing identical fatty acid profiles that were significantly different from wild type (Figure 2 and Supplementary Table 1). On average, seeds of *fad2* mutants exhibited nearly three times higher proportion of oleic acid (18:1) than wild type (35% versus 12%), whereas PUFAs content was drastically reduced. Compared to wild type, linoleic acid (18:2) content was 97% reduced (<1% versus 18%) and linolenic acid (18:3) content was 80% reduced (∼2.5% versus ∼12%). As anticipated, *fad2* seeds showed higher erucic acid (22:1) content compared to wild type (40% versus 35%) (Figure 2 and Supplementary Table 1). The observed relative changes in *Tafad2* mutants seed FA profiles are consistent with those reported in other Brassica species, including *A. thaliana* and *Camelina sativa*. These results support the concept that the loss of *fad2* function results in higher amounts of oleic acid and erucic acid content in seed TAGs due to blockage of oleic acid flux into PUFA synthesis and enhanced availability of oleic acid for elongation (Figure 1) (Jiang et al., 2017).

### CRISPR/SpCas9-Induced Mutations in the Putative *TaROD1* Gene Modestly Increase Oleic Acid Content in Pennycress Seed TAGs

To identify the putative *TaROD1* gene in pennycress, we again searched the *T. arvense* genome sequence database and the *T. arvense* transcriptome shotgun assembly, using the *A. thaliana AtROD1* coding sequence as the query. Together, these searches identified the putative *TaROD1* gene and the TSA sequence GAKE01006801.1^2^, which shared 100% nucleotide sequence identity with each other. The *TaROD1* gene is predicted to have three exons (two introns) and an open reading frame (ORF) 906 nucleotides in length sharing 85.5% nucleotide sequence identity with the 906 bp *AtROD1* ORF (AT3G15820.1) (Supplementary Figure 3).

We created a CRISPR/SpCas9 binary vector construct named TaROD1-CRISPR-Cas9_Hyg, utilizing a vector set developed by Fauser et al. (2014), which contains *Streptococcus pyogenes* Cas9 (*Sp*Cas9) coding sequences. The TaROD1-CRISPR-Cas9_Hyg vector was designed to target edits ∼176 bp downstream of the *TaROD1* translational start site (Supplementary Figure 3). T7 endonuclease screening of PCR products amplified from seven independent T_1_-generation transformants identified four of the seven plants harboring putative edits at or near the targeted *ROD1* gene site. DNA sequence analysis led us to focus on three of the T_1_ plants, which were heterozygous for either an A nucleotide insertion, a thymine (T) nucleotide insertion, or an 18 bp deletion at the CRISPR-targeted site within the *ROD1* gene. T7 endonuclease I analysis of 20 T_2_ progeny arising from these three T_1_ plants along with DNA sequence analyses and phenotypic analyses (described below) confirmed that the mutations were inherited and segregated as recessive. We named the corresponding mutant lines *rod1-3* (18 bp deletion), *rod1-4* (single A insertion), and *rod1-5* (single T insertion) (Supplementary Figure 4). Note that pennycress *rod1-1* and *rod1-2* mutant alleles, which are not part of this study, were generated by ethyl methanesulfonate (EMS) mutagenesis and were found to have seed TAG fatty acid compositions identical to those of the CRISPR-induced *rod1* mutant alleles reported here (Chopra et al., 2020).

Seeds from homozygous *rod1-3*, *rod1-4*, and *rod1-5* plants were analyzed for TAG fatty acid composition, revealing all had identical profiles which were significantly different from wild type (Figure 2 and Supplementary Table 1). These results suggest that all three mutations likely confer total loss of *ROD1* function. The *rod1-3* mutation is predicted to produce a “TGA” stop codon at the deletion site (Supplementary Figures 4A,C), whereas the *rod1-4* and *rod1-5* mutations cause frameshifts, in this case abolishing translation of 80% of the downstream portions of the ROD1 polypeptide (Supplementary Figures 4A–E). These frameshift mutations introduced premature stop codons which can cause nonsense-mediated mRNA decay – a mechanism used by cells to avoid production of truncated proteins that could be toxic (Conti and Izaurralde, 2005). On average, seeds of all *rod1* mutants had a nearly two-fold increase in oleic acid percentage (∼23% versus 12%), about a two-fold decrease in linoleic acid (∼9% versus 18%), and relatively no changes in linolenic or erucic acids content compared to wild type (Figure 2 and Supplementary Table 1). These relative fatty acid changes were comparable to those observed with *rod1* loss-of-function in *A. thaliana* and *Brassica napus*, although the magnitudes of changes were found to be different between Arabidopsis and canola (Lu et al., 2009; Bai et al., 2020).

### Stacking *fad2* or *rod1* Mutations With *fae1* Knockout Substantially Increased Seed Oil Oleic Acid Content

Wild-type pennycress seed TAGs contain about 35% erucic acid (22:1), which is produced by sequential fatty acid elongation of oleic acid (18:1) involving a protein complex containing the FA elongase enzyme, FAE1 (Haslam and Kunst, 2013; McGinn et al., 2019; Chopra et al., 2020). We reasoned that TAG oleic acid content could be increased further in the pennycress *fad2* and *rod1* knockout mutants by stacking each with an *fae1* CRISPR-induced knockout mutant we previously showed abolished erucic acid production (McGinn et al., 2019), in this way creating ultra-high (*fad2 fae1*) and high (*rod1 fae1*) oleic acid seed oil varieties. To accomplish this, we cross-pollinated *rod1* and *fad2* mutants with the *fae1-3* knockout mutant and screened for double mutants in the F_2_ generation using the above-described T7 Endonuclease method. Double homozygous *fad2-4 fae1-3*, *fad2-5 fae1-3*, and *rod1-4 fae1-3* lines were confirmed by DNA sequence analysis of gene-specific PCR products amplified from F_3_ plants. Fatty acid compositional analyses of TAGs extracted from seeds harvested from senesced plants revealed that *fad2-4 fae1-3* seed TAGs contained, on average, 91% oleic acid (compared to 12% for WT), less than 1% linoleic acid (18% for WT), less than 3% linolenic acid (12% for WT), and no erucic acid (35% for WT), whereas *rod1-4 fae1-3* seed TAGs contained about 60% oleic acid, 17% linoleic acid, 19% linolenic acid, and no erucic acid (Figure 2 and Supplementary Table 1). Both of these mutant combinations increased seed TAG oleic acid content over the 48% present in the *fae1-3* single mutant. Notably, only the *fad2-4 fae1-3* mutant combination, like the *fad2* single mutants, and not *rod1-4 fae1-3*, had greatly reduced PUFAs content.

### *fad2* Knockout Mutants Displayed Delayed Growth and Reduced Seed Yield

**FIGURE 3:**
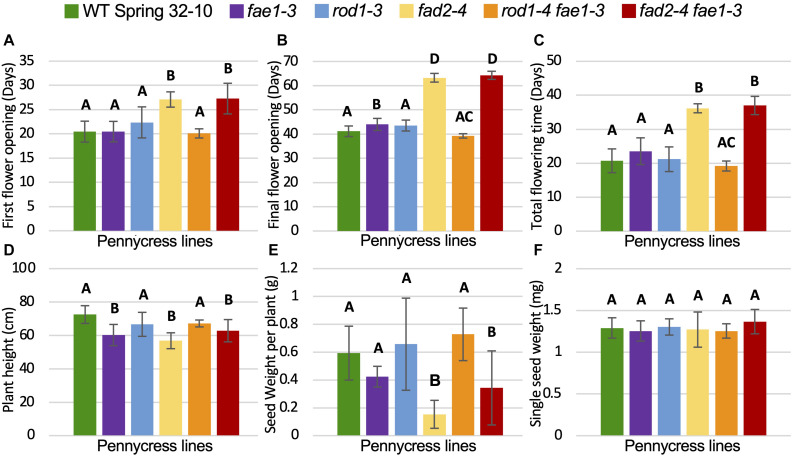
Plant growth and seed yield comparisons. **(A–F)** Graphs showing average **(A)** time until first flower opening, **(B)** time until final flower opening, **(C)** duration of flowering, **(D)** plant height upon senescence, **(E)** total weight of seeds per plant, and **(F)** single seed weight. Bars are standard deviations. Different letters above columns represent significant differences between genotypes (one-way ANOVA; Tukey test, *p* < 0.01). *n* = 11 biological reps. For **(E,F)**, all seeds from each plant were measured.

To determine if any of the mutations detailed above also affected plant growth and/or seed viability, homozygous mutant plants and wild-type controls were grown side by side in growth chambers and assessed for relative growth characteristics including morphologies, growth rates, flowering times, final plant heights, seed quantities and weights, and seed germination rates. Growth experiments were repeated at least two times for each genotype. The following data are from a representative experiment, where 12 plants per each mutant line were grown alongside wild type (see the section “Materials and Methods”). The number of days to first flower, final flower, and total flowering times were quantified along with final plant heights and seed yields (Figure 3 and Supplementary Figure 5). No obvious morphological differences were observed between the different mutant and wild-type plants. However, *fad2* and *fad2 fae1* mutant plants consistently grew much slower than wild type, whereas the other genotypes grew similar to wild type (Figures 3A–D and Supplementary Figure 5). In terms of flowering, first-flower opening for *fae1*, *rod1*, and *rod1 fae1* mutant plants occurred an average of 20.5 days post planting, similar to wild-type Spring 32-10 control plants (Figure 3A). In contrast, *fad2* and *fad2 fae1* mutant plants took, on average 6.5 days (32%) and 9.2 days (44.3%) longer to flower than wild type, respectively (Figure 3A). The days the last flowers opened also were recorded, with *fae1*, *rod1*, and *rod1 fae1* mutant plants completing flowering similarly to the 41 days taken by wild-type plants. By contrast, *fad2* and *fad2 fae1* mutant plants consistently completed flowering much later than wild type, in this case, 22 and 23 days later on average than wild type (Figure 3B and Supplementary Figure 5). Moreover, the total durations of flowering for *fad2* and *fad2 fae1* plants were significantly longer than wild-type and the other mutants (36.2 days total flowering time for *fad2*, 37 days for *fad2 fae1*, versus 20.7 days for wild type; one-way ANOVA; Tukey test, *p* < 0.01) (Figure 3C).

Final plant heights along with total amounts of seed produced and single seed weights were also quantified for all of the genotypes. Figure 3D shows that *fad2-4* and *fad2-4 fae1-3* plants grew to be significantly shorter than wild type, on average 56.8 cm for *fad2-4* and 62.8 cm for *fad2-4 fae1-3* versus 72.5 cm for wild type, representing 15–21% shorter heights (one-way ANOVA; Tukey test, *p* < 0.01). Moreover, *fad2* and *fad2 fae1* mutant plants produced significantly less seeds than wild type, on average 0.15 grams and 0.34 grams seed per *fad2-4* and *fad2-4 fae1-3* plant, respectively, versus 0.59 grams per wild-type plant (42 to 75% less; one-way ANOVA; Tukey test, *p* < 0.01) (Figure 3E). Individual seed weights were no different between the mutants and wild type (Figure 3F). For this particular experiment, *fae1-3* mutant plants grew on average to be ∼17% shorter than wild type. However, this difference was not consistently observed from experiment to experiment. Moreover, the *fae1-3* knockout mutation stacked with *rod1-4* (*rod1-4 fae1-3* plants) were on average the same height as wild type (Figure 3E), suggesting the *fae1-3* single mutant height difference may be due to factors unrelated to *fae1* loss of function, unless the *rod1* mutation is somehow compensating for the *fae1* mutation. Field studies are underway to determine what growth differences may exist between the mutants versus wild type under United States Midwest farm field conditions.

**FIGURE 4:**
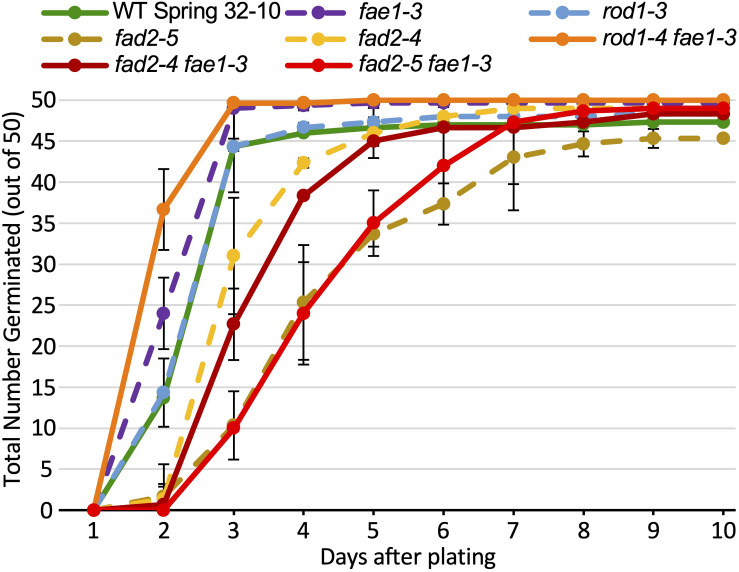
Amounts of seed germination over a 10-day period of the different lipid mutants versus wild type grown at 22°C. One hundred and fifty seeds from each line were plated on three agar growth media plates (50 seeds per plate; each plate treated as a biological rep). The data for single mutants are graphs as dashed lines, whereas double mutants and wild type (WT) are graphed as solid lines. Error bars represent standard deviations of the means. Values and significant differences can be found in Supplementary Table 2.

The seeds of the mutant genotypes and wild type also were scored for germination under various temperatures and light regimes. Described first is an experiment consisting of 150 seeds from each line spread onto three agar growth media plates (50 seeds per plate; each plate was scored as a biological rep), incubated in a growth chamber at 22°C with a 16 h/8 h light/dark cycle. Seeds were scored each day over a 10-day period for radical emergence. The data in Figure 4 and Supplementary Table 2 show that no germination occurred until 2 days after plating. After 3 days, 88% to 99% of wild-type, *rod1-3*, *fae1-3*, and *rod1-4 fae1-*3 seeds had germinated whereas only 20% to 62% of *fad2-4*, *fad2-5*, *fad2-4 fae1-3*, and *fad2-5 fae1-3* seeds had germinated. Statistical analysis of the data showed that the *fad2* genotypes germinated significantly slower than wild type (one-way ANOVA; Tukey test *p* < 0.01; Supplementary Table 2). In fact, it took an additional 2–5 days for *fad2*-containing genotypes to attain the 88% or greater amounts of germination seen for wild type and the other mutant genotypes. While significantly more *rod1-4 fae1-*3 and *fae1-3* seeds had germinated after 2 days relative to wild type (Figure 4 and Supplementary Table 2), *rod1-4* and wild type germinated similarly, putting in to question whether there is a germination benefit to *fae1* and/or *rod1* mutations. Regardless, these data show that *fae1* and *rod1* knockout mutations do not negatively affect germination under the growth conditions used. It is important to note that it is not uncommon for the germination of pennycress seeds of a given genotype including wild type to vary from batch to batch.

Plants adjust PUFA content in membranes to adapt to different temperatures, with PUFA content increasing with colder temperatures to maintain membrane fluidity (Menard et al., 2017). To see how seed germination of the different mutants might be affected by different temperatures, we performed germination assays at four different temperatures (4°C, 14°C, 22°C, and 28°C), under constant light as well as constant dark conditions. Of particular interest was how well the *fad2* mutation-containing genotypes would perform, given their severely hampered ability to produce PUFAs and the observed delays in adult plant growth. The data in Supplementary Figure 6 and Supplementary Tables 2, 3 show that seeds of all genotypes including the *fad2* single and *fad2 fae1* double mutants germinated very well at 28°C under both light and dark conditions, with most seeds germinating within 2–3 days. At 22°C, 14°C, as well as 4°C, *fad2* single mutant seeds tended to germinate slower than wild type, with the differences becoming more noticeable at the lower temperatures, especially under the constant dark conditions (Supplementary Figures 6B,D). For the most part, though, all except the *fad2* genotypes germinated comparably well to wild type under all the conditions tested. Studies are underway to determine how the various genotypes germinate and establish under farm field conditions.

### Heterogeneous Distributions of PC and TAG in Mutant and Wild-Type Seeds

To test whether spatial changes in seed lipid metabolites accompanied changes in composition, the distributions of PC and TAG molecular species were analyzed by MALDI-MS imaging of sectioned seeds harvested from senesced plants. PC is both a major structural membrane lipid as well as an important source of acyl chains that are incorporated into TAGs through the acyl editing pathway (Bates et al., 2013). Specifically, PC is the metabolite on which FAD2 adds a second double bond at the Δ12 position of an oleate moiety (Okuley et al., 1994). Hence, a disruption of the *FAD2* gene was suspected to impact the overall unsaturation of the PC pool within *fad2* containing mutants. Similarly, ROD1 acts on PC by interconverting PC and DAG by swapping the phosphocholine moiety from PC to DAG (Lu et al., 2009), which may also influence the PC molecular composition or distribution. As FAE1 acts on acyl-CoA substrates rather than on PC (James et al., 1995), the PC composition is not expected to change much in *fae1* mutants, but the length of the fatty acids in TAG is expected to change significantly.

**FIGURE 5:**
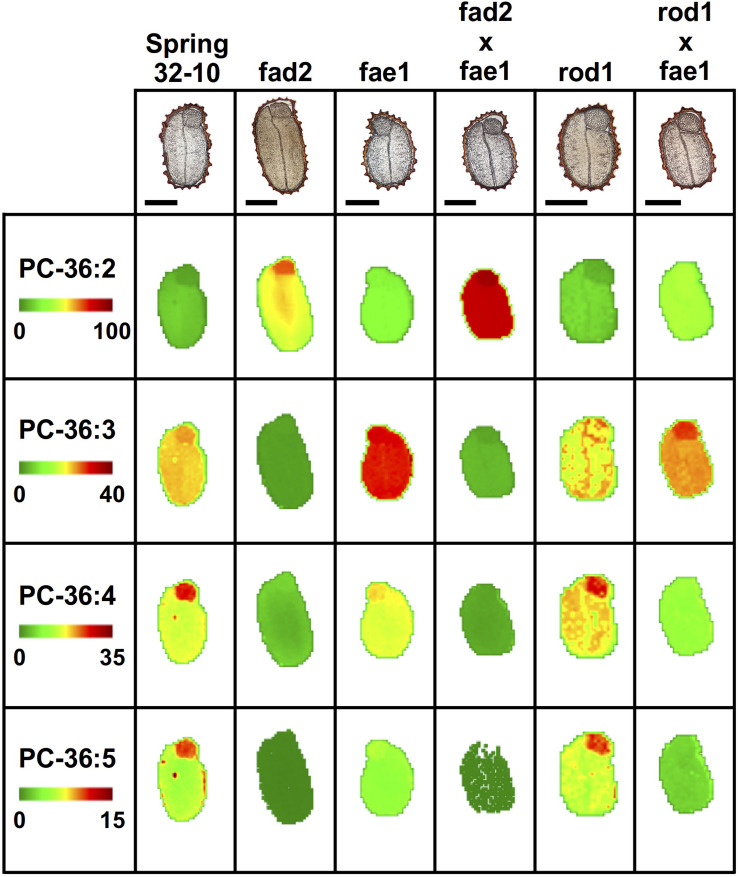
Mass spectrometry (MS) imaging of selected phosphatidylcholine (PC) molecular species in wild-type and CRISPR mutant *T. arvense* seeds. MS images are presented as false colored images with each pixel representing an individual scan rastered over the imaged seed section (microscopy image above, scale bar = 500 μm). The color intensity scale ranges from green (low) to red (high) as the mol% value of the metabolite class. Each row of images are set to the same mol% scale to show relative differences in abundance and localization between each seed type.

In the wild type, Spring 32-10, the most abundant of PC molecular species included PC-36:3 and 36:4 (Figure 5) as well as PC-34:1, 34:2, 34:3 (Supplementary Figure 7). The more unsaturated PC molecular species were primarily localized within the embryonic axis, while the less unsaturated PC species were found more enriched in the cotyledons. Disruption of the *fad2* gene had the expected result of diminishing the abundance of PC with polyunsaturated FAs and increasing the abundance of PC molecular species with more saturated FAs, such as PC-36:2 (Figure 5). While the abundance of the different PC molecular species within the single *fae1* mutant did not change much compared to wild type (Figure 5 and Supplementary Figure 7), the mutants did show an unexpected ablation of the unequal heterogeneous distribution of PC species. The double mutant *fad2 fae1* showed an expected reduction of PC molecular species diversity with PC-36:2 most dominant (Figure 5), likely containing two oleate moieties that accumulate without the action of *fad2* for further desaturation or *fae1* for elongating beyond an 18 carbon FA in acyl-CoA substrates. The single *rod1* mutant appeared to effect little change in either the abundance or distributions of PC molecular species, and the double mutant of *rod1 fae1* showed a similar abundance and distribution as the single *fae1* mutant (Figure 5 and Supplementary Figure 7).

**FIGURE 6:**
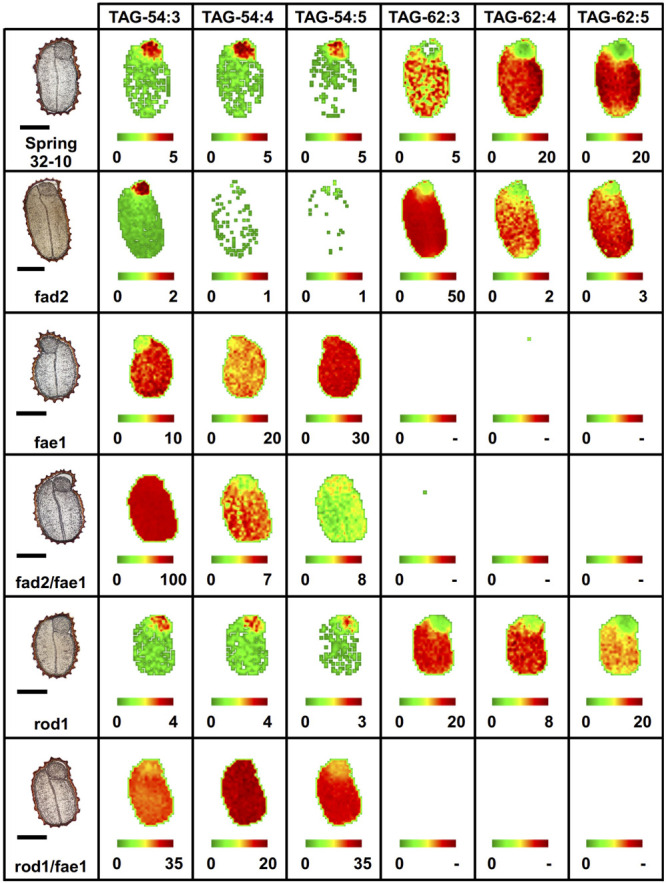
Imaging of selected triacylglycerol (TAG) molecular species (54 vs. 62 carbon TAG) in wild-type and mutant *fad2*, *fae1*, *fad2 fae1*, *rod1*, and *rod1 fae1 T. arvense* mature seed sections. MS images are presented as false colored images with each pixel representing an individual scan rastered over the imaged seed section (microscopy image left, scale bar = 500 μm). The color intensity scale ranges from green (low) to red (high) as the mol% value of the metabolite class. Each image mol% value is set individually (color scale below image) to show relative differences in abundance and localization between each seed type.

Like PC, different TAG molecular species of oilseeds have previously been observed to localize preferentially to different seed tissues (Marmon et al., 2017; Woodfield et al., 2017; Lu et al., 2018, 2020; Sturtevant et al., 2019). In wild-type seeds, the most abundant TAG molecular species were the erucic acid-containing TAG-62:4, 62:5 (Figure 6) and TAG-60:4, 60:5 (Supplementary Figure 8), preferentially localized to the cotyledons, while the embryonic axis showed a relative enrichment in 52 and 54 carbon TAG species (Supplementary Figure 8 and Figure 6). The *fad2* mutant showed similar distributions as the wild type but much less of the polyunsaturated TAG molecular species and instead an accumulation of the more saturated TAG-62:3 (Figure 6) and TAG-60:3 (Supplementary Figure 8). The *fae1* mutant showed a complete lack of TAG containing very long chain FAs, such as erucic acid, as expected without the elongation pathway. Instead, *fae1* mutants accumulated 54 carbon containing TAG in the cotyledons where previously there had been much less in the wild-type seeds (Figure 6 and Supplementary Figure 8). The added *fad2* disruption in the double mutant of *fad2 fae1* simplified the TAG molecular species present to mostly TAG-54:3 in both the cotyledons and embryonic axis uniformly, likely containing three oleic fatty acids (Figure 6).

Similar to the wild type, the *rod1* mutant showed a greater abundance of 60 and 62 carbon containing TAG, especially TAG-60:3 (Supplementary Figure 8) and TAG-62:3 (Figure 6), but showed a decrease in the more unsaturated TAG molecular species with the same number of carbons reflecting the reduced abundance of PUFAs in these mutants (Supplementary Table 1). The double mutant *rod1 fae1* showed a similar abundance and distribution of TAG molecular species as the *fae1* single mutant (Figure 6 and Supplementary Figure 8).

### Quantification of TAG and PC Content and Composition Within Wild-Type and Mutant Seeds

**FIGURE 7:**
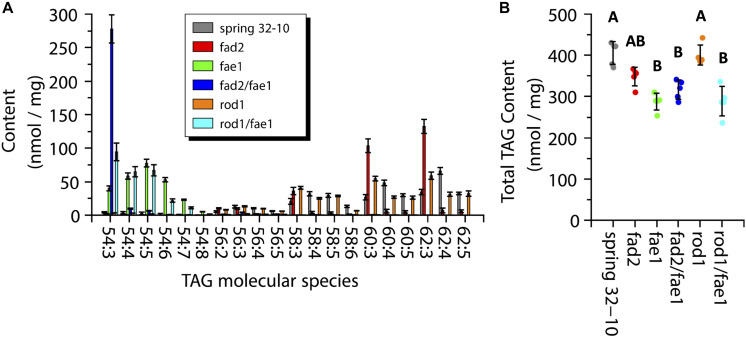
Measurements of triacylglycerol (TAG) molecular species composition **(A)** and total content **(B)** within wild-type Spring 32-10 (dark gray) and CRISPR/Cas9 mutant *T. arvense* seeds *fad2* (red), *fae1* (green), *fad2 fae1* (dark blue), *rod1* (orange), and *rod1 fae1* (light blue). Error bars represent standard deviations of the means. (*n* = 5, ±SD, one-way ANOVA; Tukey test, *p* < 0.01).

While MALDI-MS imaging is able to provide information on the spatial distribution and the relative abundance PC and TAG molecular species, it is limited in its ability to provide a quantitative measure of the amounts of these PC and TAG metabolites (Sturtevant et al., 2016). To determine the amounts of PC and TAG within the wild-type seeds and the different mutants, direct infusion ESI-MS was performed on lipid extracts made from each genotype. The total TAG measured from wild-type seeds was 405.5 ± 27.6 nmol/mg, which was nearly the same as in *rod1* seeds with 400.5 ± 24.3 nmol/mg (Figure 7B). The TAG content from *fae1* (287.7 ± 20.4 nmol/mg), *fad2 fae1* (316.8 ± 23.4 nmol/mg), and *rod1 fae1* (288.8 ± 36.0 nmol/mg) was significantly less than wild type or *rod1*, while *fad2* had an intermediate amount of TAG (348.5 ± 22.4 nmol/mg) (one-way ANOVA; Tukey test, *p* < 0.01; Figure 7B).

The major TAG molecular species found in wild-type Spring 32-10 were 60:3, 60:4, 60:5, 62:3, 62:4, and 62:5, containing the very long chain erucic acid. The two most abundant molecular species in wild-type seeds were 60:4 (48.3 ± 4.1 nmol/mg) and 62:4 (66.1 ± 5.0 nmol/mg) (Figure 7A, dark gray bars). In *fad2* seeds the TAG composition shifted to more saturated species, as seen in Figure 7A (red bars) and consistent with total FA profiles (Supplementary Table 1), with the most abundant molecular species being 60:3 (103.6 ± 10.2 nmol/mg) and 62:3 (132.5 ± 10.4 nmol/mg), which is nearly equal to the sum of all 60 and 62 carbon TAG found in wild-type seeds when including the more unsaturated species, respectively. The *fae1* seeds showed a major shift in TAG composition where the most abundant molecular species shifted to 54 carbon TAG with between three and seven double bonds in the FAs (Figure 7A, green bars). The *fad2 fae1* seeds essentially contained only the 54:3 TAG molecular species, made of three oleic fatty acids (Figure 7A, dark blue bar). The *rod1* seeds showed a similar TAG composition as wild-type seeds but with a trend toward more saturated molecular species, like *fad2* seeds with less of a stark difference (Figure 7A, orange bars). The *rod1 fae1* double mutant showed a similar composition as *fae1* seeds but with a higher content of 54:3 TAG (94.5 ± 13.1 vs. 40.1 ± 3.8 nmol/mg) (Figure 7A, light blue bars). These quantitative data from ESI-MS/MS analyses corroborated analyses of total fatty acid compositions (Supplementary Table 1) and the relative distributions determined by MALDI-MS imaging (Figure 6).

**FIGURE 8:**
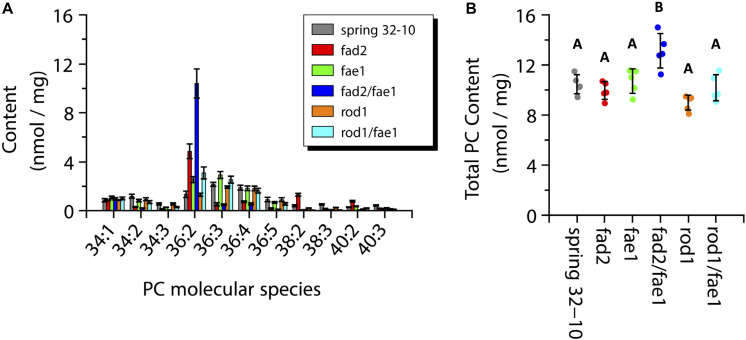
Measurements of phosphatidylcholine (PC) molecular species composition **(A)** and total PC content **(B)** within wild-type Spring 32-10 (dark gray) and CRISPR/Cas9 mutant *T. arvense* seeds *fad2* (red), *fae1* (green), *fad2 fae1* (dark blue), *rod1* (orange), and *rod1 fae1* (light blue). Error bars represent standard deviations of the means (*n* = 5, ±SD, one-way ANOVA; Tukey test, *p* < 0.01).

The total PC content across the wild-type and mutant seeds ranged from 9 to 11 nmol/mg with the exception of *fad2 fae1* seeds showing a significantly greater amount at ∼13 nmol/mg (one-way ANOVA; Tukey test, *p* < 0.01; Figure 8B). The major PC molecular species measured in wild-type seeds were the 36 carbon species containing between two and four double bonds, as well as PC-34:1 and 34:2 (Figure 8A, dark gray bars). Like TAG, the PC molecular species in *fad2* seeds shifted to more saturated species compared to the wild-type seeds, such as PC-36:2 (4.8 ± 0.6 vs. 1.3 ± 0.3 nmol/mg) and PC-38:2 (1.3 ± 0.1 vs. 0.4 ± 0.1 nmol/mg) (Figure 8A, red bars). The *fae1* seeds showed a PC molecular species composition similar to wild-type seeds with a slight increase in PC-36:2 and 36:3 (Figure 8A, green bars). The PC composition of the *fad2 fae1* double mutant was nearly entirely PC-36:2 (10.4 ± 1.2 nmol/mg) (Figure 8A, dark blue bars). Seeds from *rod1* mutants contained nearly the same composition as wild type seeds, as did *rod1 fae1* seeds with a slight increase of PC-36:2 (3.1 ± 0.5 vs. 1.3 ± 0.3 nmol/mg) (Figure 8A, orange and light blue bars, respectively). These quantitative data from ESI-MS/MS analyses corroborated analyses of total fatty acid compositions (Supplementary Table 1) and the relative distributions determined by MALDI-MS imaging (Figure 6).

## Discussion

Pennycress is being developed as an off-season oilseed-producing cash cover crop for the 80 million-acre United States Midwest Corn Belt and other temperate regions (Moser et al., 2009; Phippen and Phippen, 2012; Cubins et al., 2019; Chopra et al., 2020). As part of those efforts, we sought to determine how pennycress would respond to CRISPR/Cas9 knockout of key FA biosynthetic genes involved in FA elongation (*FAE1*), FA desaturation (*FAD2*), and trafficking of mono- and poly-unsaturated FAs between PC, DAG and TAG (*ROD1*) (Figure 1). Disruption of *FAE1*, *FAD2*, and *ROD1* function individually and in combination is known to increase the oleic acid content of seed oil in other species (Peng et al., 2010; Bates et al., 2013; Li et al., 2016; Neumann et al., 2021), although the magnitudes of changes have been found to vary from species to species, reinforcing the importance of testing hypotheses in different Brassicas instead of assuming all behave the same (Bai et al., 2020; Claver et al., 2020). This may be particularly true when comparing Brassica species having diploid genomes (e.g., *Thlaspi arvense*, *Arabidopsis thaliana*, and *Lepidium campestre*) to those having polyploid genomes (*Brassica napus*, *Camelina sativa*, and *Brassica carinata*), given that genome duplications afford the opportunity for duplicated genes to diverge in function to varying degrees (Dar et al., 2017).

Pennycress *rod1 fae1* double mutants interrogated in this study, as well as EMS-generated pennycress *rod1 fae1* double mutants reported by Chopra et al. (2020), displayed a seed TAG FA composition nearly identical to that of canola (Singh and Singh, 2010), which makes this pennycress genotype attractive commercially. Here, we also report that pennycress *fad2 fae1* double mutants produce about 90% oleic acid in the seed oil. High-oleic oil has added value for food, fuel, and industrial uses owing to its relatively lower viscosity and oxidative stability compared to genetically unimproved oils from oilseed crops. Along with compositional analyses, we sought to determine how these genetic changes might affect plant growth and health, as it is important that crop stress resilience and grain yields not be compromised by genetic changes introduced into commercial varieties. In that regard, *rod1 fae1* double mutant plants grew indistinguishable from wild type whereas *fad2* knockout mutants exhibited delayed growth (discussed below). We also documented changes to the lipid composition and spatial heterogeneity of PC and TAG species specifically within the seed via MALDI-MSI, shedding light on where different lipids are synthesized and stored in pennycress embryos derived from varying lipid metabolic pathways.

### Engineering Pennycress to Confer High Oleic Seed Oil Phenotypes

We successfully employed the *S. pyogenes* and *S. aureus* CRISPR/Cas9 systems to create mutations in the *ROD1* and *FAD2* genes, respectively. Five of the six CRISPR/Cas9-induced mutations resulted in frameshifts whereas the sixth created a premature stop codon. As such, all of the mutations in this study are likely complete loss-of-function alleles, which is supported by the fact that the mutant alleles of each gene exhibited identical phenotypes including identical FA compositional changes (Figure 2 and Supplementary Table 1). The lipid profile changes were also consistent between siblings and from generation to generation, suggesting low plasticity to these traits, at least under our controlled growth chamber conditions. It remains to be seen how plastic pennycress seed FA composition is under variable farm field conditions. Reports with other species in this regard have been mixed, with camelina varieties showing no variation in seed oil FA composition based on different weather conditions (Kurasiak-Popowska et al., 2021), whereas oilseed rape was found to have higher and lower seed oil linolenic acid content corresponding with warmer and cooler weather conditions, respectively (Wójtowicz and Wójtowicz, 2020).

Notably, PUFA content was drastically reduced in the *fad2* mutants (<5%), revealing the nearly exclusive role of *TaFAD2* in FA desaturation in pennycress seed TAGs. BLAST searches of the pennycress genome, using *TaFAD2* sequences as the query, did not identify any close *FAD2* homologs. *FAD2* in *Arabidopsis thaliana* also occurs as a single-copy gene (Beisson et al., 2003). It is possible that the small amounts of PUFAs found in seeds of *fad2* mutants were derived from the action of FAD6, a plastidial delta-12 desaturase which has similar enzymatic activity to the endoplasmic reticulum-localized FAD2 while sharing limited sequence homology (Falcone et al., 1994).

We found that pennycress *fae1* and *fad2* mutants had undesirable reductions in total amounts of seed oil compared to wild type, although those reductions varied between experiments, possibly due to differences in growing conditions. Similar relative reductions in seed oil content were reported for *Brassica napus fae1* and *fad2* RNA interference (RNAi) mutants (Shi et al., 2017). Reduced oil production in these mutants may in part be due to feedback inhibition of plastidic acetyl-CoA carboxylase (ACCase) by 18:1-acyl carrier protein (ACP), as found by Andre et al. (2012) in *Brassica napus* embryo-derived cell cultures that were fed oleic acid Tween esters. It may be possible to overcome this feedback inhibition by reducing function of the ACCase inhibiting proteins, BIOTIN ATTACHMENT DOMAIN-CONTAINING1 (BADC1) and BADC3 (Yu et al., 2021).

### Characterization of Different Plant Growth Phenotypes

While not measured in the *fad2* mutant plants’ vegetative tissues, it is possible that PUFA levels were also reduced throughout the plant. This would explain the delayed plant growth phenotypes we observed with the *fad2* knockout mutant plants (Figure 3A and Supplementary Figure 7). PUFAs are known to play important physiologic and biophysical roles, participating in signaling, regulating membrane fluidity at different temperatures, and providing resilience against environmental stressors including cold and salt stress (Browse et al., 1993; Okuley et al., 1994; Kodama et al., 1995; Weber, 2002; Zhang et al., 2012; Dar et al., 2017; Nguyen et al., 2019). Notably, linolenic acid is a precursor to the synthesis of jasmonic acid, which is a plant hormone that plays a central role in stress responses, development and growth (Huang et al., 2017). More work will be necessary to determine how these mutations affect hormone production and signaling and if the different mutants and mutant combinations are susceptible to specific abiotic and biotic stressors including drought stress and herbivory. Our germination and growth data suggest this may be the case with pennycress *fad2* loss of function. By contrast, to date, the pennycress *rod1* and *fae1* mutants and mutant combinations have performed very similar to wild type in both growth chamber (this study) and field settings (unpublished). Field conditions from fall pennycress planting to spring harvest in the United States Midwest, where these lines have been grown, are highly variable and challenging due to freeze-thaw cycles, waterlogging, and wind-blown conditions, which bodes well for the agronomic relevance of *rod1* and *fae1* mutations in improving pennycress crop characteristics. It seems likely that partial loss-of-function *fad2* mutations will also be relevant agronomically, even though our data show that total loss of *fad2* function causes agronomically unacceptable delays in growth and reduced seed yields (Figure 3 and Supplementary Figure 5). It remains to be determined what are the threshold changes in lipids composition that can be tolerated by pennycress and how undesirable phenotypes can be mitigated with compensatory genetic changes.

### Visualizing Heterogeneous Distributions of PC and TAG in Pennycress Mutant and Wild-Type Seeds via MALDI-MSI

Differences in fatty acid composition seen by GC-FID assay prompted us to investigate the distribution of the major classes of lipids in these seeds—PC and TAG. PC is an important structural membrane lipid in seeds and is also an intermediate in the biosynthesis of TAG. Given that different molecular species of PC and TAG have been found to be distributed in an unequal manner in seed tissues of many plant species (Marmon et al., 2017; Woodfield et al., 2017; Lu et al., 2018, 2020; Sturtevant et al., 2019), it was not clear how the mutant genotypes generated here would influence those normal distributions. MALDI-MS imaging allows for a comprehensive view of the lipid metabolites distributed in seed sections. Here, the spatial distribution for different molecular species of PC and TAG were visualized, and these mutations in enzymes of seed lipid metabolism influenced these metabolite distributions in some surprising ways. In wild-type seeds, the more unsaturated PC species containing 34 and 36 fatty acid carbons were localized mostly within the embryonic axis, while those that were more saturated were found more throughout the cotyledons (Supplementary Figures 7, 8). This distribution was largely the same in the *rod1* mutant. The *fad2* seeds retained some heterogeneous separation between the cotyledonary and embryonic axis tissues, most easily seen with PC-34:1 (Supplementary Figure 7), but with a somewhat less diverse PC lipid profile. However, *fae1*, *fad2 fae1*, and *rod1 fae1* revealed a lack of heterogeneity in seed tissues, suggesting the mutations made in *FAE1* were most impactful on pathways that would otherwise establish that heterogeneity observed in wild-type seeds. Likewise, many of the distributions of 54 carbon TAG species in these same three mutants were uniform across the seed tissues (Figure 6), reinforcing the precursor-product relationship between acyl groups in PC and those in TAG. The 54 carbon TAG species became the major molecular species in *fae1*-containing mutant seeds, made up of 18 carbon fatty acids with varying levels of unsaturation between the three mutants, with *fad2 fae1* being the most saturated (i.e., TAG-54:3 with 18:1/18:1/18:1) and the single *fae1* mutant being the most unsaturated. Among all the wild-type and mutant seeds, those TAG molecular species that were lower molecular weight, such as 52 carbon, but also 54 and 56 carbon species in wild type, *fad2*, and *rod1*, were primarily enriched in the embryonic axis and less abundant, relatively speaking, in the cotyledons (Supplementary Figure 8), suggesting that elongation of fatty acids normally predominates in cotyledonary tissues and that this drives the unequal accumulation of erucic-acid containing TAGs in these parts of the seed. The loss of heterogeneity observed in *fae1*, *fad2 fae1*, and *rod1 fae1* most likely is attributed to the mutation in *FAE1*, as it is common to all three mutant genotypes, and *FAE1* has been found to be primarily expressed in cotyledonary tissues in developing seeds (Rossak et al., 2001; Lu et al., 2020). Lipid species containing VLCFAs, like those produced via FAE1, have previously been found to accumulate in cotyledonary tissues of seeds (Horn et al., 2013; Woodfield et al., 2017; Lu et al., 2020). The interruption of *FAE1* in the pennycress mutant seeds may result in remaining metabolic pathways for TAG accumulations in cotyledons to more resemble what is found in the embryonic axis tissues, resulting in less heterogeneous distributions of both PC and TAG molecular species.

### Quantifying the Changes in PC and TAG Profiles in Pennycress Wild-Type and Mutant Seeds

Most of the mutations made here in this study produced results within PC and TAG profiles that were expected: *fad2* produced more saturated lipid species, likely one double bond in every fatty acid of either PC or TAG; *fae1* resulted in a shifted TAG profile from those containing very long chain fatty acids (56, 58, 60, 62) to those containing largely 18 carbon fatty acids (54), but had little effect on PC; the double mutant *fad2 fae1* reduced the diversity of PC and TAG to largely PC-36:2 and TAG-54:3; *rod1* resulted in slightly more saturated TAG species, though much more subtly than *fad2*, and having little effect on PC; and *rod1 fae1* with a TAG profile most similar to the single *fae1* mutant, but with an increase in TAG-54:3, and overall little effect on the PC profile (Figures 7, 8). One interesting set of mutants were those containing an interruption in ROD1. Though ROD1 is involved with PC/DAG interconversion, neither the PC composition nor total content differed greatly with the wild-type pennycress seeds. As desaturation occurs on PC, it might be expected that ROD1 would have a greater effect on the level of unsaturation in TAG, as DAG could then become interconverted to PC and become more unsaturated, and, indeed, *rod1* mutants tended to have more saturated TAG, but the overall effect of the mutation was fairly modest (Figure 7). The PC and TAG distributions as revealed by MALDI-MS imaging and the total TAG content between the single *rod1* mutant and wild-type seeds were nearly identical (Figures 5, 6). Likely this was a reflection of the contribution of LPCAT as a route for the release of PUFA-CoAs, and their incorporation into TAG by the Kennedy pathway (Figure 1); or by cholinephosphotransferase (CPT) as a partially redundant activity for the interconversion of PC and DAG in the absence of *rod1*. The mutants that appeared to show the greatest changes in TAG composition and content were those containing an interruption in FAE1. The three mutants *fae1, fad2 fae1*, and *rod1 fae1* all showed a significantly lower amount of TAG compared to either wild type or the single mutant *rod1*, whereas *fad2* contained a TAG content intermediate between wild type and its double mutant with *fae1* (Figure 7). This may suggest FAE1 exerts a much greater influence on the total TAG content and composition stored within pennycress seeds, which in wild type seeds are notable for accumulating a high amount of erucic acid (22:1). Future engineering efforts will need to overcome this obstacle when creating new pennycress lines with altered TAG compositions in order to create not only pennycress seed oils with oil compositions more suitable to their intended applications, such as drop-in fuels, but also high oil producing lines.

## Conclusion

Pennycress is proving to be a useful model system for translational biology research, allowing for comparative analyses and the implementation of powerful genetics tools such as CRISPR/Cas9 gene editing for rapid and efficient production of mutants and corresponding genotypic and phenotypic analyses. The different pennycress single and double mutants that were generated for this study displayed unique plant and seed phenotypes captured in a variety of characterization methods, the data of which will be useful in guiding future studies of mutant performance under different stressors in both laboratory and field conditions. Here, we also provide data showing that pennycress *rod1 fae1* double mutations confer a lipid profile similar to that of canola seed TAGs. The pennycress *rod1 fae1* mutant combination appears to be an attractive genotype for commercialization into food, feed, and fuel products including biodiesel given that these genotypes grow as well as wild type, although more growth tests remain to be performed to determine how resilient the genotypes are to a variety of abiotic and biotic stresses. Furthermore, this study sheds light on tissue-specific localizations of PC and TAG molecular species within pennycress embryos and how lipid metabolic fluxes may be affected by various mutations in the targeted fatty acid modifying genes, hinting at useful metabolic studies and related genetic changes aimed at optimizing lipid compositions while also enhancing total oil yields and avoiding disruption of important physiologic functions (Sedbrook and Durrett, 2020; Tsogtbaatar et al., 2020).

## Materials and Methods

### Surface Sterilization of Pennycress Seeds and Growth Conditions

Pennycress seeds were surface sterilized with a brief rinse of 70% ethanol followed by a 10-min incubation in a sterilization solution consisting of 30% bleach and 0.01% SDS. After sterilization solution removal, the seeds were rinsed three times with sterile water. Gibberellin treatment was not necessary for Spring 32 seed germination as it has low primary seed dormancy.

Surface-sterilized pennycress seeds were sown onto “agar growth media,” consisting of 0.8% agar media containing one-half-strength Murashige and Skoog salts in petri dishes wrapped in Parafilm. Immediately after seed sowing, the plates were placed into a Percival Scientific CU-36L5 incubator (16 h 4100K fluorescent light ∼150–200 μE/m^2^/s/8 h dark, 22°C). For growth in soil, seedlings were transplanted at a density of four plants per 4-inch pot (Gage Dura Pots 4″ × 3–3/8″, OBC Northwest Inc. catalog no. PPG4) in autoclaved Miracle Grow soil mix (or a 50/50 mix of Redi-Earth plug and seedling mix and Berger BM 7 bark mix) intermixed with 0.03 g/4-inch pot of the insecticide Marathon (Marathon 1% Granular^3^). When making up the 4-inch pots with Redi-Earth and BM 7 soils, a thin layer of wet soil (∼1/4 inch) was first put in the bottom of the pot, on top of which 1/8 teaspoon of prilled urea (46-0-0; Greenway Biotech, Inc.) was sprinkled before the pot was entirely filled with the wet soil mix. For the flowering time assays, time zero was when seedlings were transplanted into soil. Plants were grown in environment-controlled growth chambers cycling 16 h light/8 h dark (light was either 6500K fluorescent or a combination 4100K fluorescent/incandescent lighting, 175–250 μE/m^2^/s light intensity), at 21 or 22°C.

### Vectors

The TaFAD2_CRISPR-Sa-Cas9_Hyg and TaROD1_CRISPR-Sp-Cas9_Hyg binary vectors were constructed and generated, as described in Fauser et al. (2014); Steinert et al. (2015), and at http://www.botanik.kit.edu/molbio/940.php, using the vectors pEn-Sa-Chimera and pDe-Sa-Cas9 or pEn-Sp-Chimera and pDe-Sp-Cas9, respectively. The plant selectable marker (Bar gene) in the pDe-sa-Cas9 binary vector was replaced with the Hygromycin phosphotransferase (hpt) gene (40 U/ml hygromycin selection in plants) to create a pDe-Sa-Cas9_Hyg and pDe-Sp-Cas9_Hyg vectors. Bacterial selection used for the binary vector was 75 μg/ml spectinomycin.

The following two oligos were annealed to create the 20-mer protospacer specific to the open reading frame of the putative pennycress *TaFAD2* gene. Note that the nucleotides ATTG on the FWD primer and CAAT on the REV primer serve as overhangs for annealing to the vector’s *Bsa*I restriction sites:

PennyFAD2_CRISPR_FWD: ATTGTACTTGGCTTGGCCTCTCTA
PennyFAD2_CRISPR_REV: TAGAGAGGCCAAGCCAAGTACAAT

The following two oligos were annealed to create the 20-mer protospacer specific to the open reading frame of the putative pennycress *TaROD1* gene:

PennyROD1_CRISPR_FWD: ATTGGACGACGGCTACGCAAACGG
PennyROD1_CRISPR_REV: CCGTTTGCGTAGCCGTCGTCCAAT

Constructs were introduced into *Agrobacterium tumefaciens* strain GV3101 using a standard CaCl_2_ flash-freeze/thaw transformation method (Holsters et al., 1978).

### *Agrobacterium*-Mediated Transformation of Pennycress

Cultures of *Agrobacterium tumefaciens* strain GV3101 containing either the TaFAD2-CRISPR-SaCas9_Hyg or TaROD1-CRISPR-SpCas9_Hyg plasmid were grown from glycerol stocks (∼200 uL inoculated into 50 mL Luria Broth (LB) containing 50 μg/ml gentamycin, 50 μg/ml rifampicin, and 75 μg/ml spectinomycin. The 50 mL cultures were shaken overnight at 28°C, then added to an additional 200 mL LB antibiotic-containing media and again incubated overnight, then centrifuged at 3,500 RPM for 10 min and resuspended in an equal volume of 5% (w/v) sucrose plus 0.02% (v/v) Silwet L-77. The floral portion of inflorescences of plants that had flowers opening for ∼5 days were submerged in this *Agrobacterium* solution, then placed under ∼30 inches mercury (14.7 psi) vacuum in a 26 cm × 25 cm × 36.5 cm vacuum chamber for 10 min, using a diaphragm vacuum pump (60 L/Min pump speed, 200 mBar ultimate vacuum). After dipping, the floral portions of the inflorescences were wrapped in plastic wrap sealed around the stems with twist ties, and the plants placed back into an environmental growth chamber. The plastic wrap covering was removed the following day.

### Identification of Transgenic and CRISPR-Cas9-Edited Pennycress Plants

#### Antibiotic Selection

Putative TaFAD2-CRISPR-SaCas9_Hyg or TaROD1-CRISPR_SpCas9 transgenic seeds were surface sterilized and plated onto 0.8% agar/one half-strength Murashige and Skoog salts containing 40 U/mL *hygromycin* B. Seedlings that continued to grow on the antibiotic-containing media were transferred to soil ∼8 days after plating, then after establishment were further confirmed as being transgenic by PCR analysis.

#### Screening for CRISPR/Cas9 Edits: T7 Endonuclease I

Leaf-extracted genomic DNA was PCR amplified using *FAD2* or *ROD1* primers spanning the CRISPR-Cas9 target site (see Supplementary Materials), followed by T7 endonuclease I digestion of the PCR product as described by Pyott et al. (2016). Template for PCR reactions was a 50:50 mix of each putative mutant prep and wild type to ensure that even in the case of a homozygous mutation, a DNA mismatch would be PCR amplified and detected. PCR template was extracted from fresh leaf tissue using the Phire Plant Direct PCR Kit (Thermo #F130WH). For T7 endonuclease I analysis, 10 μl of each 20 μl PCR reaction was denatured by heating at 95°C for 5 min in a thermocycler (Fisher) and annealed using gradual cooling: −2°C per second decrease from 95°C to 85°C then −0.1°C per second decrease from 85°C to 25°C, followed by T7 endonuclease I (Fisher Scientific cat. #50-995-224) digestion for 30 min. The digested product was electrophoresed in a 1% agarose gel to identify samples that partially digested, indicating an SaCas9 or SpCas9-induced edit in *TaFAD2* or *TaROD1* genes, respectively, which were confirmed by Sanger DNA sequence analyses of PCR products amplified from cetyltrimethyl ammonium bromide (CTAB)-purified genomic DNA (Clarke, 2009).

### Characterization Assays

#### Germination Studies

Seeds were surface sterilized and plated onto 0.8% agar/one half-strength Murashige and Skoog salts (agar growth media) with no antibiotics and no sucrose. Plates were observed at the same time daily for radical emergence until all seeds germinated or 16 days. Seed germination times were compared to wild type using one-way ANOVA; Tukey test, *p* < 0.01. *n* = 150 seeds split in to three biological reps (50-seed replicates plated on to agar growth media).

#### Flowering Times

Plants were observed and scored at a daily time for the opening of the first flower on their inflorescences. The same plants were also observed and scored daily at the same time for the final flower to open. These time points were compared to the time of transplant and to each other for total flower time window.

#### Plant Heights

Final plant heights were recorded by cutting the plants at the base by the soil and measuring the length from the base to the top of the main stem, straightening the stem against a measuring stick.

#### Seed Analysis Data

Quantifications of numbers of seeds, total seed weights per plant, and single-seed weight of the different CRISPR mutants and wild type were performed using a MARViTECH MARViN 5.0 Seed Analyzer instrument along with an Ohaus STX223 balance (Single-seed weight = total seed weight/number of seeds).

#### Lipid Analysis

Total lipids were extracted from pennycress seeds harvested from senesced plants, and fatty acid methyl esters (FAMEs) were generated and analyzed by gas chromatography, as described in Cahoon et al. (2006). For ESI-MS analysis, neutral lipids were purified from total seed lipids on a small silica column with 99:1 (v/v) chloroform: methanol. Samples were then analyzed on an API4000 triple quadrupole mass spectrometer (Applied Biosystems) as described previously (Bansal and Durrett, 2016). To quantify fatty acid composition, total seed lipids were separated using thin layer chromatography on Silica gel 60 plates (Merck) with a 70:30:1 hexane:diethyl-ether:acetic acid (v/v/v) solvent system. Lipids were visualized by spraying with 0.075% 20,70 -dichlorofluorescein in 95% methanol and exposing to UV light. The bands were scraped and directly transmethylated using a base-catalyzed method (Ichihara et al., 1996); the resulting FAMEs were analyzed using gas chromatography.

### MALDI-MS Imaging of Pennycress Seed Sections

Pennycress seeds harvested from senesced plants were sectioned then imaged by MALDI-MS imaging using methods as previously described by Rolletschek et al. (2020). Data collected from MS imaging experiments used the same parameters described previously, and were analyzed in a similar manner with the exception that PC images were derived from the sum of the [M + H]^+^ and [M + K]^+^ adducts, and TAG as the sum of the [M + Na]^+^ and [M + K]^+^ adducts.

### ESI-MS Quantification of PC and TAG From Lipid Extracts of Pennycress Seeds

Total lipids from whole seeds (10–15 mg) were extracted into CHCl_3_ using procedures previously described (Sturtevant et al., 2019). Lipid extracts were diluted by 1:50 into 1:1:0.01 CHCl_3_/MeOH/5 mM NH_4_Ac prior to analysis. Diluted extracts were analyzed via direct infusion ESI-MS using a Waters Synapt G2-Si mass spectrometer (Waters Corporation, Milford, MA, United States). The mass spectrometer and direct infusion parameters were set as follows: an *m/z* range of 600–1200, source temperature of 80°C, desolvation gas set to 500 L/h, capillary voltage set to 3 kV, and an injection rate of 20 μl/min. Data was collected with MassLynx software (Waters Corporation, Milford, MS, United States). A mixture of PC and TAG standards consisting of tripentadecanoin (15:0/15:0/15:0, 1.05 μM), tripalmitin (16:0/16:0/16:0, 0.99 μM), triheptadecanoin (17:0/17:0/17:0, 0.94 μM), tristearin (18:0/18:0/18:0, 1.01 μM), triolein (18:1/18:1/18:1, 1.02 μM), trilinolein (18:2/18:2/18:2, 1.02 μM), trilinolenin (18:3/18:3/18:3, 1.03 μM), triheneicosanoin (21:0/21:0/21:0, 0.98 μM), dimyristoylphosphocholine (PC-14:0/14:0, 0.1 μM), diheptadecanoylphosphocholine (PC-17:0/17:0), and diheneicosanoylphosphocholine (PC-21:0/21:0, 0.1 μM) was used to determine the correction factors for carbon number and unsaturations for ESI-MS data. TAG standards were synthesized by Nu-Chek Prep. Inc. (Elysian, MN, United States), and the PC standards were purchased from Sigma Aldrich (St. Louis, MO, United States; PC-14:0/14:0: ca. no. P2663; PC-17:0/17:0: ca. no. 850360C, PC-21:0/21:0: ca. no. 850370P). Relative correction factors were calculated from the intensities of each standard and then plotted against either the number of fatty acid carbons or number of fatty acid double bonds. The equation for the fitted linear trend line was used to calculate the adjustment factors for each potential number of carbon or double bond found in the detected TAG and PC molecular species of the pennycress lipid extracts. The product of the two correction factors (carbon no. and double bond number) was multiplied by the calculated nmol/mg of TAG or PC detected to produce the final corrected amount. Samples were spiked with 1 nmol of dimyristoylphosphocholine and 1 nmol of triheptadecanoin as internal standards prior to analysis.

## Data Availability Statement

The original contributions presented in the study are included in the article/Supplementary Material, further inquiries can be directed to the corresponding author.

## Author Contributions

BJ and MM generated the CRISPR/Cas9 constructs and mutants. BJ genotypically and phenotypically characterized the CRISPR/Cas9 mutants. TN performed the fatty acids analyses. TR performed MALDI-MS imaging and ESI-MS/MS quantification of PC and TAG. BJ, MM, TN, EC, KC, and JS designed and interpreted experiments. BJ, TR, KC, and JS wrote and edited the manuscript. All authors contributed to the article and approved the submitted version.

## Conflict of Interest

Illinois State University has entered a licensing agreement with CoverCress, Inc. for use of the *fae1* germplasm.

## Footnotes

1 https://www.ncbi.nlm.nih.gov/nuccore/%20GAKE01001774.1

2 https://www.ncbi.nlm.nih.gov/nuccore/%20GAKE01006801.1

3 www.domyown.com/

## Supplementary Material

Supplementary Figure 1 Nucleotide sequence alignment of the *AtFAD2* and *TaFAD2* ORFs.

Supplementary Figure 2 T7 endonuclease analysis and sequences of the *fad2* mutants.

Supplementary Figure 3 Nucleotide sequence alignment of the *AtROD1* and *TaROD1* ORFs.

Supplementary Figure 4 Sequences of the *rod1* mutants.

Supplementary Figure 5 Pictures of mutants and wild type growth over time.

Supplementary Figure 6 Graphed amounts of seed germination over a 16-day period under different temperatures and light regimes.

Supplementary Figure 7 MS imaging of PC molecular species detected in wild-type and mutant embryos.

Supplementary Figure 8 MS imaging of TAG molecular species detected in wild-type and mutant embryos.

Supplementary Table 1 Seed TAG fatty acid compositions of the different mutants.

Supplementary Table 2 Total seed germination on agar growth media plates.

Supplementary Table 3 Amounts of seed germination in constant light at different temperatures.

Supplementary Table 4 Amounts of seed germination in constant darkness at different temperatures.
