# Supplementary material for: CRISPR/Cas9-Induced fad2 and rod1 Mutations Stacked With fae1 Confer High Oleic Acid Seed Oil in Pennycress (Thlaspi arvense L.)
Source: Front Plant Sci. 2021 Apr 22;12:652319. doi: 10.3389/fpls.2021.652319 (PMC8100250; doi:10.3389/fpls.2021.652319)
Supplement: Supplementary Figure 1 — Nucleotide sequence alignment of the AtFAD2 and TaFAD2 ORFs. [file Data_Sheet_1.PDF]

frontiers

Supplemental Material

CRISPR/Cas9-Induced *fad2* and *rod1* Mutations Stacked with *fae1* Confer High Oleic Acid Seed Oil in Pennycress (*Thlaspi arvense* L.)

Brice A. Jarvis[1], Trevor B. Romsdahl[2], Michaela McGinn[1], Tara J. Nazarenus[3], Edgar B. Cahoon[3], Kent D. Chapman[2], and John C. Sedbrook[1*]

[1]School of Biological Sciences, Illinois State University, Normal, IL 61790, USA

[2]BioDiscovery Institute and Department of Biological Sciences, University of North Texas, Denton, TX 76203, USA

[3]Department of Biochemistry and Center for Plant Science Innovation, University of Nebraska-Lincoln, Lincoln, NE 68588 USA

***Correspondence:** John Sedbrook, Email: jcsedbr@ilstu.edu

Table S1. Seed TAG fatty acid compositions of the different pennycress CRISPR-induced oilseed mutants. Values represent means plus/minus standard deviations. Asterisks represent significant differences compared to wild type based on Student t-test analysis where $p < 0.05$. n = 3. These data are graphed in Figure 2.

Genotype	16:0	16:1	18:0	18:1	18:2	18:3	20:1	22:1	24:1
WT Spring 32-10	3.0±0.1	0.3±0.0	0.4±0.0	12.1±0.8	18.1±0.5	11.7±0.4	12.0±0.5	34.9±0.9	3.3±0.1
fad2-4 (-2bp)	2.1±0.1**	0.1±0.1**	0.4±0.0	34.9±0.7**	0.5±0.2**	1.7±0.1**	16.6±0.4**	39.3±0.7**	3.4±0.1**
fad2-5 (+A)	2.4	0.1	0.4	35.0	0.5	2.5	14.9	39.8	3.8
fad2-6 (-29bp)	2.5	0.1	0.4	34.0	0.5	2.7	14.2	41.4	3.6
rod1-3 (-18bp)	3.4±0.4*	0.4±0.0**	0.6±0.0**	22.7±1.0**	8.5±0.6**	11.8±0.6	14.0±0.3**	35.3±2.0	2.8±0.2**
rod1-4 (+A)	3.2±0.3	0.4±0.0**	0.5±0.0**	22.4±0.7**	9.1±0.8**	11.2±0.2	14.4±0.2**	35.7±1.5	3.0±0.3
rod1-5 (+T)	4.3	0.0	0.5	23.1	10.5	12.5	14.3	31.7	2.8
fae1-3 (-4bp)	3.5±0.1**	0.3±0.0	0.8±0.0**	47.8±1.9**	28.5±1.4**	17.8±0.6**	0.9±0.1**	0.0±0.0**	0.3±0.0**
fad2-4 fae1-3	2.5±0.2**	0.2±0.1	0.6±0.0**	90.6±0.6**	0.5±0.1**	2.6±0.3**	1.5±0.1**	0.0±0.0**	0.2±0.2**
rod1-4 fae1-3	3.4±0.0*	0.0±0.0**	0.6±0.0**	59.7±0.3**	17.1±0.2*	19.3±0.3**	0.0±0.0**	0.0±0.0**	0.0±0.0**

Table S2. Total seed germination on agar growth media plates of the different pennycress CRISPR-induced oilseed mutants. Values represent cumulative germination over a 10-day period (total means of 50). Standard deviations represent plus/minus the average daily new germination. Asterisks represent significant differences compared to wild type based on one-way ANOVA; Tukey test analysis versus wild type where $p < 0.05$* and $p < 0.01$**. n=3 biological reps of 50 seeds each. These data are graphed in Figure 4.

	**Day 1**	**Day 2**	**Day 3**	**Day 4**	**Day 5**	**Day 6**	**Day 7**	**Day 8**	**Day 9**	**Day 10**
WT Spring32-10	0.0±0.0	13.7±4.0	44.3±5.7	46.0±1.5	46.7±0.6	47.0±0.6	47.0±0.0	47.0±0.0	47.3±0.6	47.3±0.0
fae1-3 (-4bp)	0.0±0.0	24.0±4.4*	49.0±5.2	49.3±0.6	49.7±0.6	49.7±0.0	49.7±0.0	49.7±0.0	49.7±0.0	49.7±0.0
rod1-4 (+A)	0.0±0.0	14.3±4.2	44.3±5.6	46.7±0.6	47.3±0.6	48.0±1.2	48.0±0.0	48.0±0.0	48.3±0.6	48.3±0.0
fad2-5 (+A)	0.0±0.0	1.7±1.5**	10.3±7.1**	25.3±0.6**	33.7±3.1*	37.3±1.7**	43.0±1.0	44.7±0.0	45.3±0.0	45.3±0.0
fad2-4 (-2bp)	0.0±0.0	1.3±1.5**	31.0±4.2**	42.3±7.0	46.0±1.5	48.0±2.5	49.0±6.4	49.0±1.5	49.0±1.2	49.0±0.0
rod1-3 fae1-3	0.0±0.0	36.7±4.9**	49.7±4.4	49.7±0.0	50.0±0.6	50.0±0.0	50.0±0.0	50.0±0.0	50.0±0.0	50.0±0.0
fad2-4 fae1-3	0.0±0.0	0.7±0.6**	22.7±1.7**	38.3±0.6	45.0±1.2	46.7±1.5*	46.7±0.0	47.3±0.6	48.3±1.0	48.3±0.0
fad2-5 fae1-3	0.0±0.0	0.0±0.0**	10.0±0.0**	24.0±6.2**	35.0±4.0*	42.0±5.0**	44.0±2.0	45.3±1.5	45.7±0.6	45.7±0.0

Table S3. Amounts of seed germination in constant light at different temperatures over a 16-day period. Data can be found in graph form in Figure S5. Values are the means of the number of seeds that germinated on the given day plus/minus standard deviations. 3 biological reps of 25 seeds each rep. Student T-test versus wild type, $p < 0.05$*, $p < 0.01$**.

**WT Spring32-10**	**Day 1**	**Day 2**	**Day 3**	**Day 4**	**Day 5**	**Day 6**	**Day 7**	**Day 8**	**Day 9**	**Day 10**	**Day 11**	**Day 12**	**Day 13**	**Day 14**	**Day 15**	**Day 16**
**4 ºC**	0.0±0.0	0.0±0.0	0.0±0.0	0.0±0.0	0.0±0.0	0.0±0.0	0.0±0.0	0.0±0.0	0.0±0.0	0.7±0.6	0.7±1.2	1.3±2.3	0.3±0.6	0.0±0.0	0.0±0.0	0.0±0.0
**12 ºC**	0.0±0.0	0.0±0.0	0.0±0.0	14.3±8.5	8.3±6.7	1.7±1.5	0.0±0.0	0.0±0.0	0.3±0.6	0.0±0.0	0.0±0.0	0.0±0.0	0.0±0.0	0.0±0.0	0.0±0.0	0.0±0.0
**22 ºC**	0.0±0.0	11.3±6.0	9.7±6.7	2.7±0.6	1.00±1.0	0.3±0.6	0.0±0.0	0.0±0.0	0.0±0.0	0.0±0.0	0.0±0.0	0.0±0.0	0.0±0.0	0.0±0.0	0.0±0.0	0.0±0.0
**28 ºC**	0.0±0.0	14.7±3.1	7.0±2.6	1.0±1.0	0.0±0.0	0.3±0.6	0.3±0.6	0.0±0.0	0.0±0.0	0.67±1.2	0.33±0.6	0.0±0.0	0.0±0.0	0.0±0.0	0.0±0.0	0.0±0.0
fae1-3																
**4 ºC**	0.0±0.0	0.0±0.0	0.0±0.0	0.0±0.0	0.0±0.0	0.3±0.6	0.0±0.0	0.0±0.0	0.3±0.6	0.0±0.0	0.3±0.0	1.0±1.7	0.3±0.6	0.0±0.0	0.3±0.6	0.3±0.6
**12 ºC**	0.0±0.0	0.0±0.0	1.7±2.9	9.7±6.1	10.0±6.6	2.7±2.3	0.0±0.0	0.3±0.6	0.0±0.0	0.0±0.0	0.0±0.0	0.3±0.6	0.0±0.0	0.0±0.0	0.0±0.0	0.0±0.0
**22 ºC**	0.0±0.0	16.0±0.0	7.7±0.6	1.00±1.0*	0.0±0.0	0.0±0.0	0.0±0.0	0.0±0.0	0.33±0.6	0.0±0.0	0.0±0.0	0.0±0.0	0.0±0.0	0.0±0.0	0.0±0.0	0.0±0.0
**28 ºC**	0.0±0.0	12.0±5.6	7.3±3.5	4.7±4.0	0.7±1.2	0.3±0.6	0.0±0.0	0.0±0.0	0.0±0.0	0.0±0.0	0.0±0.0	0.0±0.0	0.0±0.0	0.0±0.0	0.0±0.0	0.0±0.0
rod1-3																
**4 ºC**	0.0±0.0	0.0±0.0	0.0±0.0	0.0±0.0	0.0±0.0	0.0±0.0	0.0±0.0	0.3±0.6	0.7±1.2	0.7±1.2	0.0±0.0	0.0±0.0	0.0±0.0	0.0±0.0	0.0±0.0	0.0±0.0
**12 ºC**	0.0±0.0	0.0±0.0	3.3±5.8	7.3±7.1	10.3±9.6	3.7±3.2	0.3±0.6	0.0±0.0	0.0±0.0	0.0±0.0	0.0±0.0	0.0±0.0	0.0±0.0	0.0±0.0	0.0±0.0	0.0±0.0
**22 ºC**	0.0±0.0	17.7±3.2	5.0±3.6	1.3±2.3	0.0±0.0	0.0±0.0	0.0±0.0	0.0±0.0	0.0±0.0	0.0±0.0	0.0±0.0	0.0±0.0	0.0±0.0	0.0±0.0	0.0±0.0	0.0±0.0
**28 ºC**	0.0±0.0	10.3±5.5	10.3±4.2	3.00±1.0	1.00±1.0	0.0±0.0	0.0±0.0	0.0±0.0	0.0±0.0	0.0±0.0	0.0±0.0	0.0±0.0	0.0±0.0	0.0±0.0	0.0±0.0	0.0±0.0
fad2-5																
**4 ºC**	0.0±0.0	0.0±0.0	0.0±0.0	0.0±0.0	0.0±0.0	0.0±0.0	0.0±0.0	0.0±0.0	0.0±0.0	0.7±1.2	0.0±0.0	0.3±0.6	0.0±0.0	0.3±0.6	0.3±0.6	0.0±0.0
**12 ºC**	0.0±0.0	0.0±0.0	0.0±0.0	2.7±2.5*	6.0±3.0	5.7±1.5	3.0±2.0	1.0±0.0	0.3±0.6	1.0±1.0	0.7±0.6	0.3±0.6	0.0±0.0	0.0±0.0	0.0±0.0	0.0±0.0
**22 ºC**	0.0±0.0	3.3±2.1	10.0±6.0	7.0±1.0*	1.3±1.5	0.7±1.2	2.0±1.0*	0.3±0.6	0.0±0.0	0.0±0.0	0.0±0.0	0.0±0.0	0.0±0.0	0.0±0.0	0.0±0.0	0.0±0.0
**28 ºC**	0.0±0.0	11.3±0.6	6.3±3.8	4.3±3.2	0.7±0.6	0.7±1.2	0.3±0.6	0.3±0.6	0.3±0.6	0.0±0.0	0.0±0.0	0.0±0.0	0.0±0.0	0.0±0.0	0.0±0.0	0.0±0.0
rod1-4 fae1-3																
**4 ºC**	0.0±0.0	0.0±0.0	0.0±0.0	0.0±0.0	0.0±0.0	0.0±0.0	0.0±0.0	0.3±0.6	0.0±0.0	0.3±0.6	1.0±1.7	0.7±1.2	0.7±1.2	0.0±0.0	0.0±0.0	0.0±0.0
**12 ºC**	0.0±0.0	0.0±0.0	2.7±4.6	11.3±2.3	10.3±7.2	0.0±0.0	0.0±0.0	0.0±0.0	0.0±0.0	0.3±0.6	0.0±0.0	0.0±0.0	0.0±0.0	0.0±0.0	0.0±0.0	0.0±0.0
**22 ºC**	0.0±0.0	16.3±2.3	8.0±2.0	0.7±1.2*	0.0±0.0	0.0±0.0	0.0±0.0	0.0±0.0	0.0±0.0	0.0±0.0	0.0±0.0	0.0±0.0	0.0±0.0	0.0±0.0	0.0±0.0	0.0±0.0
**28 ºC**	0.0±0.0	1.3±0.6*	5.3±3.2	10.7±4.5*	4.0±3.0	2.0±1.0*	1.0±1.0	0.3±0.6	0.0±0.0	0.0±0.0	0.0±0.0	0.0±0.0	0.0±0.0	0.0±0.0	0.0±0.0	0.0±0.0
fad2-4 fae1-3																
**4 ºC**	0.0±0.0	0.0±0.0	0.0±0.0	0.0±0.0	0.0±0.0	0.0±0.0	0.0±0.0	0.0±0.0	0.0±0.0	0.3±0.6	0.7±1.2	0.7±1.2	1.3±1.2	1.7±2.1	0.3±0.6	0.0±0.0
**12 ºC**	0.0±0.0	0.0±0.0	3.0±5.2	8.7±2.1	8.0±4.0	1.7±2.1	1.3±1.5	0.7±0.6	1.0±0.0	0.3±0.6	0.0±0.0	0.0±0.0	0.0±0.0	0.0±0.0	0.0±0.0	0.0±0.0
**22 ºC**	0.0±0.0	14.7±0.6	6.0±3.5	3.3±3.1	1.0±1.0	0.0±0.0	0.0±0.0	0.0±0.0	0.0±0.0	0.0±0.0	0.0±0.0	0.0±0.0	0.0±0.0	0.0±0.0	0.0±0.0	0.0±0.0
**28 ºC**	0.0±0.0	16.3±4.7	4.0±2.0	2.7±1.2	0.3±0.6	0.7±1.2	0.0±0.0	0.0±0.0	0.0±0.0	0.0±0.0	0.0±0.0	0.0±0.0	0.0±0.0	0.0±0.0	0.0±0.0	0.0±0.0

Table S4. Amounts of seed germination in constant darkness at different temperatures over a 16-day period. Data can be found in graph form in Figure S5. Values are the means of the number of seeds that germinated on the given day plus/minus standard deviations. 3 biological reps of 25 seeds each rep. Student T-test versus wild type, p<0.05*, p<0.01**.

WT Spring32-10	Day 1	Day 2	Day 3	Day 4	Day 5	Day 6	Day 7	Day 8	Day 9	Day 10	Day 11	Day 12	Day 13	Day 14	Day 15	Day 16
4 ºC	0.0±0.0	0.0±0.0	0.0±0.0	0.0±0.0	0.0±0.0	0.0±0.0	0.0±0.0	1.0±0.0	0.7±1.2	4.0±2.6	3.7±4.0	1.7±2.9	4.3±3.1	0.7±0.6	1.0±1.7	0.7±1.2
12 ºC	0.0±0.0	0.0±0.0	2.7±4.6	5.7±9.0	8.0±6.2	7.0±6.1	1.0±1.0	0.3±0.6	0.0±0.0	0.0±0.0	0.0±0.0	0.0±0.0	0.0±0.0	0.0±0.0	0.0±0.0	0.0±0.0
22 ºC	0.0±0.0	16.0±3.5	6.7±1.5	1.0±0.0	0.0±0.0	0.3±0.6	0.0±0.0	0.3±0.6	0.0±0.0	0.0±0.0	0.33±0.0	0.0±0.0	0.0±0.0	0.0±0.0	0.0±0.0	0.0±0.0
28 ºC	0.0±0.0	15.3±4.2	6.7±5.9	0.7±1.2	0.0±0.0	0.00±0.0	0.0±0.0	0.3±0.6	0.0±0.0	1.0±1.0	0.0±0.0	0.0±0.0	0.0±0.0	0.0±0.0	0.0±0.0	0.0±0.0
fae1-3																
4 ºC	0.0±0.0	0.0±0.0	0.0±0.0	0.0±0.0	0.0±0.0	0.0±0.0	0.0±0.0	1.3±0.6	0.3±0.0	4.7±3.2	9.0±2.0	0.7±1.2	4.3±2.9	0.7±0.6	0.7±0.6	0.0±0.0
12 ºC	0.0±0.0	0.0±0.0	6.7±11.5	4.0±3.0	7.7±5.8	6.3±6.0	0.3±0.6	0.0±0.0	0.0±0.0	0.0±0.0	0.0±0.0	0.0±0.0	0.0±0.0	0.0±0.0	0.0±0.0	0.0±0.0
22 ºC	0.0±0.0	18.3±2.3	5.3±2.1	0.7±0.6	0.0±0.0	0.3±0.6	0.0±0.0	0.0±0.0	0.0±0.0	0.0±0.0	0.0±0.0	0.0±0.0	0.0±0.0	0.0±0.0	0.0±0.0	0.0±0.0
28 ºC	0.0±0.0	15.7±3.8	5.0±2.6	1.0±1.0	1.3±1.2	0.0±0.0	0.3±0.6	0.0±0.0	0.0±0.0	0.3±0.6	0.0±0.0	0.0±0.0	0.0±0.0	0.0±0.0	0.0±0.0	0.0±0.0
rod1-3																
4 ºC	0.0±0.0	0.0±0.0	0.0±0.0	0.0±0.0	0.0±0.0	0.0±0.0	0.0±0.0	0.0±0.0	0.7±0.6	2.0±2.6	3.3±3.5	2.7±4.6	0.3±0.6	0.7±0.6	0.7±0.6	0.0±0.0
12 ºC	0.0±0.0	0.0±0.0	4.7±8.1	3.3±5.8	7.3±5.7	5.7±5.5	2.0±2.6	1.3±2.3	0.0±0.0	0.7±1.2	0.0±0.0	0.0±0.0	0.0±0.0	0.0±0.0	0.0±0.0	0.0±0.0
22 ºC	0.0±0.0	18.7±5.0	5.7±5.1	0.3±0.6	0.3±0.6	0.0±0.0	0.0±0.0	0.0±0.0	0.0±0.0	0.0±0.0	0.0±0.0	0.0±0.0	0.0±0.0	0.0±0.0	0.0±0.0	0.0±0.0
28 ºC	0.0±0.0	14.0±6.2	9.3±5.5	0.7±0.6	0.3±0.6	0.0±0.0	0.0±0.0	0.0±0.0	0.0±0.0	0.0±0.0	0.0±0.0	0.0±0.0	0.0±0.0	0.0±0.0	0.0±0.0	0.0±0.0
fad2-5																
4 ºC	0.0±0.0	0.0±0.0	0.0±0.0	0.0±0.0	0.0±0.0	0.0±0.0	0.0±0.0	0.3±0.6	0.0±0.0	0.0±0.0	1.0±1.0	0.0±0.0	0.7±0.6	0.7±1.2	0.0±0.0	0.0±0.0
12 ºC	0.0±0.0	0.0±0.0	0.0±0.0	3.3±4.9	2.3±2.1	5.3±2.1	4.33±1.5**	1.7±1.5	2.7±0.6**	0.3±0.6	1.0±1.0	1.0±1.7	0.0±0.0	0.0±0.0	0.0±0.0	0.0±0.0
22 ºC	0.0±0.0	4.0±4.4*	9.3±2.1	6.7±3.8	1.7±1.2	1.0±1.0	0.7±1.2	0.7±1.2	0.0±0.0	0.0±0.0	0.0±0.0	0.0±0.0	0.0±0.0	0.0±0.0	0.0±0.0	0.0±0.0
28 ºC	0.0±0.0	17.7±2.1	3.0±0.0	1.7±2.1	1.0±1.0	0.0±0.0	0.0±0.0	0.7±0.6	0.0±0.0	0.7±0.6	0.0±0.0	0.0±0.0	0.0±0.0	0.0±0.0	0.0±0.0	0.0±0.0
rod1-4 fae1-3																
4 ºC	0.0±0.0	0.0±0.0	0.0±0.0	0.0±0.0	0.0±0.0	0.0±0.0	0.0±0.0	0.0±0.0	0.7±1.2	4.0±1.7	6.3±2.3	1.3±2.3	6.7±5.0	2.0±1.0	0.7±0.6	0.7±1.2
12 ºC	0.0±0.0	0.0±0.0	7.0±12.1	1.3±2.3	6.0±5.3*	9.7±8.7	0.7±1.2	0.3±0.6	0.0±0.0	0.0±0.0	0.0±0.0	0.0±0.0	0.0±0.0	0.0±0.0	0.0±0.0	0.0±0.0
22 ºC	0.0±0.0	15.7±1.2	6.7±1.2	2.3±0.6	0.3±0.6	0.0±0.0	0.0±0.0	0.0±0.0	0.0±0.0	0.0±0.0	0.0±0.0	0.0±0.0	0.0±0.0	0.0±0.0	0.0±0.0	0.0±0.0
28 ºC	0.0±0.0	6.0±2.6*	10.3±2.9	5.3±5.9	1.0±1.0	1.7±1.2	0.3±0.6	0.0±0.0	0.0±0.0	0.0±0.0	0.0±0.0	0.0±0.0	0.0±0.0	0.0±0.0	0.0±0.0	0.0±0.0
fad2-4 fae1-3																
4 ºC	0.0±0.0	0.0±0.0	0.0±0.0	0.0±0.0	0.0±0.0	0.3±0.0	2.0±0.0	0.7±0.0	1.3±0.0	1.3±0.0	0.7±0.0	1.0±0.0	4.3±0.0	2.0±0.0	0.0±0.0	1.0±0.0
12 ºC	0.0±0.0	0.0±0.0	5.3±0.0	2.7±0.0	5.3±0.0	7.7±0.0	1.0±0.0	1.0±0.0	0.3±0.0	0.0±0.0	0.0±0.0	0.0±0.0	0.0±0.0	0.0±0.0	0.0±0.0	0.0±0.0
22 ºC	0.0±0.0	16.3±0.0	6.0±0.0	2.0±0.0	0.7±0.0	0.0±0.0	0.0±0.0	0.0±0.0	0.0±0.0	0.0±0.0	0.0±0.0	0.0±0.0	0.0±0.0	0.0±0.0	0.0±0.0	0.0±0.0
28 ºC	0.0±0.0	22.7±0.0*	1.3±0.0	0.3±0.0	0.0±0.0	0.3±0.0	0.0±0.0	0.3±0.0	0.0±0.0	0.0±0.0	0.0±0.0	0.0±0.0	0.0±0.0	0.0±0.0	0.0±0.0	0.0±0.0

Martinez/Needleman-Wunsch DNA Alignment
Minimum Match: 9; Gap Penalty: 1.10; Gap Length Penalty: 0.33

Seq1(1>1152) TaFAD2 ORF GAKE01001774.seq	Seq2(1>1152) AtFAD2 ORF AT3G12120.seq	Similarity Index	Gap Number	Gap Length	Consensus Length
(1>1152)	(1>1152)	88.8	0	0	1152

```
         v10       v20       v30       v40       v50       v60       v70       v80       v90
ATGGGTGCAGGTGGAAGAATGACGGTTCCTACTTCTTCCAAGAAGTCTGAAACCGATGCCTTAAAGCGTGTGCCGTGCGAGAAACCGCCG
ATGGGTGCAGGTGGAAGAATG CGGTTCCTACTTCTTCCAAGAA TC GAAACCGA  CC  AAAGCGTGTGCCGTGCGAGAAACCGCC
ATGGGTGCAGGTGGAAGAATGCCGGTTCCTACTTCTTCCAAGAAATCGGAAACCGACACCACAAAGCGTGTGCCGTGCGAGAAACCGCCT
         ^10       ^20       ^30       ^40       ^50       ^60       ^70       ^80       ^90
         v100      v110      v120      v130      v140      v150      v160      v170      v180
TTCACGCTCGGAGAACTGAAGAAAGCAATCCCACAGCATTGTTTCAATCGCTCAATCCCTCGCTCTTTCTCCTACCTTATCTGGGACATC
TTC CG T GGAGA CTGAAGAAAGCAATCCC C GCATTGTTTCAA CGCTCAATCCCTCGCTCTTTCTCCTACCTTATC G GACATC
TTCTCGGTGGGAGATCTGAAGAAAGCAATCCCGCCGCATTGTTTCAAACGCTCAATCCCTCGCTCTTTCTCCTACCTTATCAGTGACATC
         ^100      ^110      ^120      ^130      ^140      ^150      ^160      ^170      ^180
         v190      v200      v210      v220      v230      v240      v250      v260      v270
ATCATAGCCTCTTGCTTCTACTACGTTGCCACCACTTACTTCTCTCTCCTCCCTCAGCCTCTCTCTTACTTGGCTTGGCCTCTCTATTGG
AT ATAGCCTC TGCTTCTACTACGT GCCACCA TTACTTCTCTCTCCTCCCTCAGCCTCTCTCTTACTTGGCTTGGCC CTCTATTGG
ATTATAGCCTCATGCTTCTACTACGTCGCCACCAATTACTTCTCTCTCCTCCCTCAGCCTCTCTCTTACTTGGCTTGGCCACTCTATTGG
         ^190      ^200      ^210      ^220      ^230      ^240      ^250      ^260      ^270
         v280      v290      v300      v310      v320      v330      v340      v350      v360
GTCTGTCAAGGCTGTGTCTTAACCGGAGTCTGGGTCATAGCTCACGAATGCGGCCACCACGCCTTCAGCGACTACCAATGGCTTGACGAC
G CTGTCAAGGCTGTGTC TAAC GG  TCTGGGTCATAGC CACGAATGCGG CACCACGC TTCAGCGACTACCAATGGCT GA GAC
GCCTGTCAAGGCTGTGTCCTAACTGGTATCTGGGTCATAGCCCACGAATGCGGTCACCACGCATTCAGCGACTACCAATGGCTGGATGAC
         ^280      ^290      ^300      ^310      ^320      ^330      ^340      ^350      ^360
         v370      v380      v390      v400      v410      v420      v430      v440      v450
ACAGTCGGTCTGATCTTCCATTCTTTCCTCCTCGTCCCTTACTTCTCCTGGAAATACAGCCACCGCCGTCACCATTCCAACACCGGATCA
ACAGT GGTCT ATCTTCCATTC TTCCTCCTCGTCCCTTACTTCTCCTGGAA TA AG CA CGCCGTCACCATTCCAACAC GGATC
ACAGTTGGTCTTATCTTCCATTCCTTCCTCCTCGTCCCTTACTTCTCCTGGAAGTATAGTCATCGCCGTCACCATTCCAACACTGGATCC
         ^370      ^380      ^390      ^400      ^410      ^420      ^430      ^440      ^450
         v460      v470      v480      v490      v500      v510      v520      v530      v540
CTTGAAAAGGACGAAGTGTTTGTCCCTAAACAGAAATCCGCCATCAAATGGTACGGCAAGTACCTCAACAACCCTCTGGGACGCACCGTG
CT GAAA  GA GAAGT TTTGTCCC AA CAGAAATC GC ATCAA TGGTACGG AA TACCTCAACAACCCTCT GGACGCA C TG
CTCGAAAGAGATGAAGTATTTGTCCCAAAGCAGAAATCAGCAATCAAGTGGTACGGGAAATACCTCAACAACCCTCTTGGACGCATCATG
         ^460      ^470      ^480      ^490      ^500      ^510      ^520      ^530      ^540
         v550      v560      v570      v580      v590      v600      v610      v620      v630
ATGTTAACCGTCCAGTTCACCCTTGGCTGGCCCTTGTACTTAGCCTTCAACGTCTCGGGGAGACCCTACGACGGGTTCGCTTGCCACTTC
ATGTTAACCGTCCAGTT   CCT GG TGGCCCTTGTACTTAGCCTT AACGTCTC GG AGACC TA GACGGGTTCGCTTGCCA TTC
ATGTTAACCGTCCAGTTTGTCCTCGGGTGGCCCTTGTACTTAGCCTTTAACGTCTCTGGCAGACCGTATGACGGGTTCGCTTGCCATTTC
         ^550      ^560      ^570      ^580      ^590      ^600      ^610      ^620      ^630
         v640      v650      v660      v670      v680      v690      v700      v710      v720
CACCCAAACGCTCCCATCTACAACGACCGTGAACGCCTCCAGATATACATCTCGGATGCTGGTATCCTCGCCGTCTGTTACGGTCTCTAC
  CCC AACGCTCCCATCTACAA GACCG GAACGCCTCCAGATATAC TCTC GATGC GGTAT CT GCCGTCTGTT  GGTCT TAC
TTCCCCAACGCTCCCATCTACAATGACCGAGAACGCCTCCAGATATACCTCTCTGATGCGGGTATTCTAGCCGTCTGTTTTGGTCTTTAC
         ^640      ^650      ^660      ^670      ^680      ^690      ^700      ^710      ^720
         v730      v740      v750      v760      v770      v780      v790      v800      v810
CGTTACGCTGCTGCACAAGGAGTGGCCTCGATGATCTGCGTCTACGGAGTTCCGCTTCTGATAGTCAACGGGTTCCTCGTCTTGATCACA
CGTTACGCTGCTGCACAAGG  TGGCCTCGATGATCTGC TCTACGGAGT CCGCTTCTGATAGT AA G GTTCCTCGTCTTGATCAC
CGTTACGCTGCTGCACAAGGGATGGCCTCGATGATCTGCCTCTACGGAGTACCGCTTCTGATAGTGAATGCGTTCCTCGTCTTGATCACT
         ^730      ^740      ^750      ^760      ^770      ^780      ^790      ^800      ^810
         v820      v830      v840      v850      v860      v870      v880      v890      v900
TACTTGCAGCACACCCATCCCTCGTTGCCTCACTACGATTCATCCGAGTGGGATTGGTTCAGGGGAGCTTTGGCTACCGTAGACAGAGAC
TACTTGCAGCACAC CATCCCTCGTTGCCTCACTACGATTCATC GAGTGGGA TGG TCAGGGGAGCTTTGGCTACCGTAGACAGAGAC
TACTTGCAGCACACTCATCCCTCGTTGCCTCACTACGATTCATCAGAGTGGGACTGGCTCAGGGGAGCTTTGGCTACCGTAGACAGAGAC
         ^820      ^830      ^840      ^850      ^860      ^870      ^880      ^890      ^900
         v910      v920      v930      v940      v950      v960      v970      v980      v990
TATGGAATCCTGAACAAGGTCTTCCACAACATCACGGACACGCACGTGGCTCACCACCTGTTCTCGACGATGCCGCATTACCATGCGATG
TA GGAATC TGAACAAGGT TTCCACAACAT AC GACAC CACGTGGCTCA CACCTGTTCTCGAC ATGCCGCATTA  A GC ATG
TACGGAATCTTGAACAAGGTGTTCCACAACATTACAGACACACACGTGGCTCATCACCTGTTCTCGACAATGCCGCATTATAACGCAATG
         ^910      ^920      ^930      ^940      ^950      ^960      ^970      ^980      ^990
         v1000     v1010     v1020     v1030     v1040     v1050     v1060     v1070     v108
GAGGCCACGAAGGCGATAAAGCCGATACTCGGGGACTATTACCAGTTTGATGGAACACCGGTCTTCAAGGCGATGTGGAGGGAGGCGAAG
GA GC AC AAGGCGATAAAGCC AT CT GG GACTATTACCAGTT GATGGAACACCG   T     GCGATGT  AGGGAGGC AAG
GAAGCTACAAAGGCGATAAAGCCAATTCTGGGAGACTATTACCAGTTCGATGGAACACCGTGGTATGTAGCGATGTATAGGGAGGCAAAG
         ^1000     ^1010     ^1020     ^1030     ^1040     ^1050     ^1060     ^1070     ^108
         v1090     v1100     v1110     v1120     v1130     v1140     v1150
GAGTGTGTCTATGTAGAACCGGACAGGAAAGGTGAGAAGAAAGGTGTGTTCTGGTACAACAACAAGTTGTGA
GAGTGT TCTATGTAGAACCGGACAGG AAGGTGA AAGAAAGGTGTGT CTGGTACAACAA AAGTT TGA
GAGTGTATCTATGTAGAACCGGACAGGGAAGGTGACAAGAAAGGTGTGTACTGGTACAACAATAAGTTATGA
         ^1090     ^1100     ^1110     ^1120     ^1130     ^1140     ^1150
```

Figure S1. Nucleotide sequence alignment of the *Thlaspi arvense TaFAD2* ORF (top sequence) versus the *Arabidopsis thaliana AtFAD2* ORF (AT3G12120.1). The *TaFAD2* ORF sequence is derived from transcriptome assembly contig GAKE01001774.1 (Dorn et al., 2014), which shares 100% identity with *Thlaspi arvense* MN106 reference genome sequences. The red line delineates the 20 nucleotide protospacer sequence used in the CRISPR/SaCas9 construct. Outlined in blue is the NNGGGT protospacer adjacent motif (PAM) recognized by SaCas9.

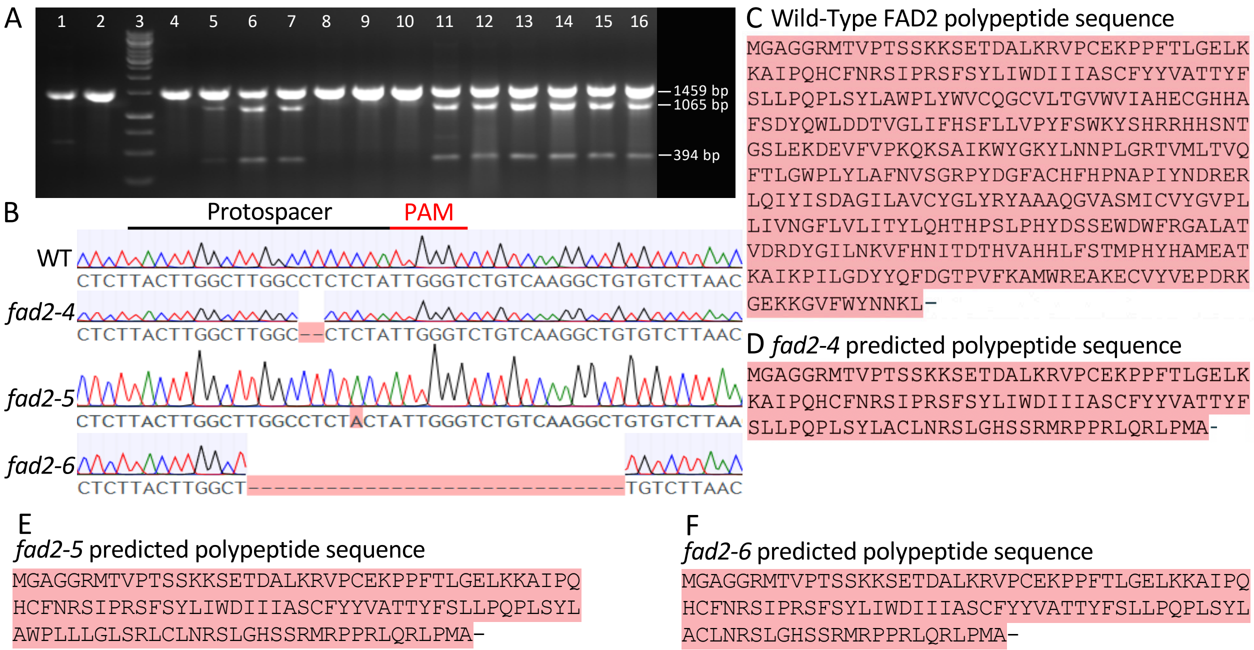

Figure S2. Characterization of the CRISPR-induced *Thlaspi arvense fad2* mutants' sequences. (**A**) Electrophoresed T7 endonuclease I-digested PCR products scoring wild type (WT) *FAD2* (1459 bp band) versus *fad2* mutations (partial digestion of 1459 bp band producing 1065 and 394 bp products). Lanes 1, 2, 4, 8-10 are WT segregants, whereas lanes 5-7 and 11-16 harbor the *fad2-3* mutation. Lane 3: Fermentas 1kb GeneRuler. (**B**) DNA sequence chromatograms. WT *TaFAD2* (top); *fad2-4* allele (2 bp deletion); *fad2-5* allele (A insertion); *fad2-6* allele (29 bp deletion). All three mutations are located as expected within the protospacer binding site of the CRISPR/*Sa*Cas9 guide RNA. (**C, D**) Predicted FAD2 polypeptide sequence in (**C**) WT pennycress versus the (**D**) *fad2-4* **(E)** *fad2-5*, and **(F)** *fad2-6* mutants. Note the predicted polypeptides encoded by each mutant are predicted to be truncated due to the frameshift mutations in the open reading frame (ORF).

Martinez/Needleman-Wunsch DNA Alignment
Minimum Match: 9; Gap Penalty: 1.10; Gap Length Penalty: 0.33

Seq1(1>906) TaROD1 ORF GAKE01006801.seq	Seq2(1>906) AtROD1 ORF AT3G15820.seq	Similarity Index	Gap Number	Gap Length	Consensus Length
(1>906)	(10>906)	85.5	4	15	909

```
         v10       v20       v30       v40       v50       v60       v70       v80       v90
ATGTCAACTAAAACCGTCGTCCCTCTCCGTCGCAGATCTAAGCCCCTTAACGGAAATCACACTAACGGCGTCGCCATTGACGGAAGCCTC
    CA CT AAACCG CGTC CTCTCCGTCGCAGATCTAA  C CTTAACGGAAA CACACTAACGGCGTCGCCATTGACGGAA CCT
GCCGCAGCTGAAACCGACGTCTCTCTCCGTCGCAGATCTAACTCTCTTAACGGAAACCACACTAACGGCGTCGCCATTGACGGAACCCTA
^10       ^20       ^30       ^40       ^50       ^60       ^70       ^80       ^90
         v100      v110      v120      v130      v140      v150      v160      v170
GACGACGACCACAACCGTCGCATCGGATCAGTAAATAGCCAAATGGATAACATTGCTAAGAAAACGGACGACGGCTACGCAAACGG---C
   GAC AC ACAACCGTCGC TCGGA     AAA A  CA ATGGATA    TGCTAAGAAAAC GAC ACGGCTACGC AA GG   C
---GACAACAACAACCGTCGCGTCGGAGATACAAACACTCACATGGATATATCTGCTAAGAAAACTGACAACGGCTACGCCAATGGTGTC
   ^100      ^110      ^120      ^130      ^140      ^150      ^160      ^170      ^180
  v180      v190      v200      v210      v220      v230      v240      v250      v260
GGAGGAGGAGGAGGAGGAGGGAAAAGCAAGGCGTCGTTTATGACGTGGACGGCGCGTGACGTTGTGTACGTGGCGAGGTACCATTGGATA
GGAGGAGGAGGA       GGA AAGCAA GCGTCGTT A GACGTGGACGGCGCGTGA  T GT TACGTGG GAG TACCATTGGATA
GGAGGAGGAGGA------TGGAGAAGCAAAGCGTCGTTCACGACGTGGACGGCGCGTGATATCGTCTACGTGGTGAGATACCATTGGATA
   ^190            ^200      ^210      ^220      ^230      ^240      ^250      ^260      ^270
  v270      v280      v290      v300      v310      v320      v330      v340      v350
CCGTGTTTGTTCGCGGTCGGGGTTCTGTTCTTCACGGGCGTGGAGTACACGCTCCAGATGATTCCCGCGAGGTCTGAGCCGTTCGATATT
CCGTG  TGTTCGC G CGG  TTCTGTTCTTCA GGGCGTGGAGTACACGCT CAGATGATTCCCGCGAG TCTGAGCCGTTCGAT TT
CCGTGCATGTTCGCTGCCGGACTTCTGTTCTTCATGGGCGTGGAGTACACGCTTCAGATGATTCCCGCGAGATCTGAGCCGTTCGATCTT
         ^280      ^290      ^300      ^310      ^320      ^330      ^340      ^350      ^360
  v360      v370      v380      v390      v400      v410      v420      v430      v440
GGGTTTGTGGCCACGCGCTCTCTGAATCGCGTCTTGGCAAATTCACCGGATCTTAACACCGTCTTAGCCGCTCTAAACACGGTGTTCGTA
GGGTTTGTGG CACGCGCTCT TGAATCGCGT TT GCA  TTCACCGGATCTTAACAC GT TTAGCCGC CTAAACACGGTGTTCGTA
GGGTTTGTGGTCACGCGCTCTTTGAATCGCGTATTAGCATCTTCACCGGATCTTAACACTGTTTTAGCCGCACTAAACACGGTGTTCGTA
         ^370      ^380      ^390      ^400      ^410      ^420      ^430      ^440      ^450
  v450      v460      v470      v480      v490      v500      v510      v520      v530
GGGATGCAAACGACGTATATTGTATGGACATGGTTAATGGAAGGACGACCACGAGCCACCATCTCGGCTTGCTTCATGTTTACTTGTCGA
GGGATGCAAAC ACGTATATTGTATGGACATGGTTA TGGAAGGACGA CACGAGCCACCATC CGGCTT  TTCATGTT ACTTGTCG
GGGATGCAAACAACGTATATTGTATGGACATGGTTAGTGGAAGGACGAGCACGAGCCACCATCGCGGCTTTATTCATGTTCACTTGTCGC
         ^460      ^470      ^480      ^490      ^500      ^510      ^520      ^530      ^540
  v540      v550      v560      v570      v580      v590      v600      v610      v620
GGCATTCTTGGTTACTCTACTCAGCTCCCTCTTCCTCAGGATTTTCTAGGATCAGGTGTCGATTTTCCGGTGGGAAACGTCTCGTTCTTC
GGCATTCT GG TACTCTACTCAGCT CCTCT CCTCAGGA TTTCTAGGATCAGG GT GATTTTCCGGTGGGAAA GTCTC TTCTTC
GGCATTCTCGGCTACTCTACTCAGCTTCCTCTCCCTCAGGACTTTCTAGGATCAGGGGTTGATTTTCCGGTGGGAAATGTCTCTTTCTTC
         ^550      ^560      ^570      ^580      ^590      ^600      ^610      ^620      ^630
  v630      v640      v650      v660      v670      v680      v690      v700      v710
CTCTTCTACTCGGGTCACGTCGCCGGTTCGATGATCGCATCTTTGGACATGAGGAGAATGCAGAGGATGAGACTAGCGATGCTTTTTGAC
CTCTTCT CTC GG CA GTCGCCGG TCGATGATCGCATC TTGGACATGAG AGAATGCAGAGG TGAGACT GC ATG T TTTGAC
CTCTTCTTCTCTGGCCATGTCGCCGGCTCGATGATCGCATCATTGGACATGAGAAGAATGCAGAGGTTGAGACTTGCAATGGTCTTTGAC
         ^640      ^650      ^660      ^670      ^680      ^690      ^700      ^710      ^720
  v720      v730      v740      v750      v760      v770      v780      v790      v800
ATCCTCAATGTATTACAATCGATCAGGCTGCTCGGGACGAGAGGACACTACACGATTGATCTCGCTGTCGGAGTTGGCGCTGGGATTCTC
ATCCTCAATGTATTACA TCGATCAG CTGCTCGG AC AGAGGACA TACAC AT GA CT GC GT GGAGTTGGCGCTGGGATTCTC
ATCCTCAATGTATTACAGTCGATCAGACTGCTCGGTACAAGAGGACATTACACAATCGACCTTGCGGTTGGAGTTGGCGCTGGGATTCTC
         ^730      ^740      ^750      ^760      ^770      ^780      ^790      ^800      ^810
  v810      v820      v830      v840      v850      v860      v870      v880      v890
TTTGATTCATTCGCCGGCAAGTACGAAGAGATGATAAGCAAGAGACACAATTTAGTCAATGGTTTTGGTTTGATTTCGAAAGACTCGCTA
TT GA TCATT GCCGG AAGTACGAAGAGATGAT AGCAAGAGACA   TTTAG CA TGGTTTT GTTTGATTTCGAAAGACTC CTA
TTCGACTCATTGGCCGGAAAGTACGAAGAGATGATGAGCAAGAGACA---TTTAGGCACTGGTTTTAGTTTGATTTCGAAAGACTCTCTA
         ^820      ^830      ^840      ^850         ^860      ^870      ^880      ^890
  v900
GTCAATTAA
GTCAATTAA
GTCAATTAA
  ^900
```

Figure S3. Nucleotide sequence alignment of the *Thlaspi arvense TaROD1* ORF (top sequence) versus the *Arabidopsis thaliana AtROD1* ORF (AT3G15820.1). The *TaROD1* ORF sequence was derived from transcriptome assembly contig GAKE01006801.1 (Dorn et al., 2014), which shares 100% identity with *Thlaspi arvense* MN106 reference genome sequences. The red line delineates the 20 nucleotide protospacer sequence used in the CRISPR/SpCas9 construct. Outlined in blue is the NGG protospacer adjacent motif (PAM) recognized by SpCas9.

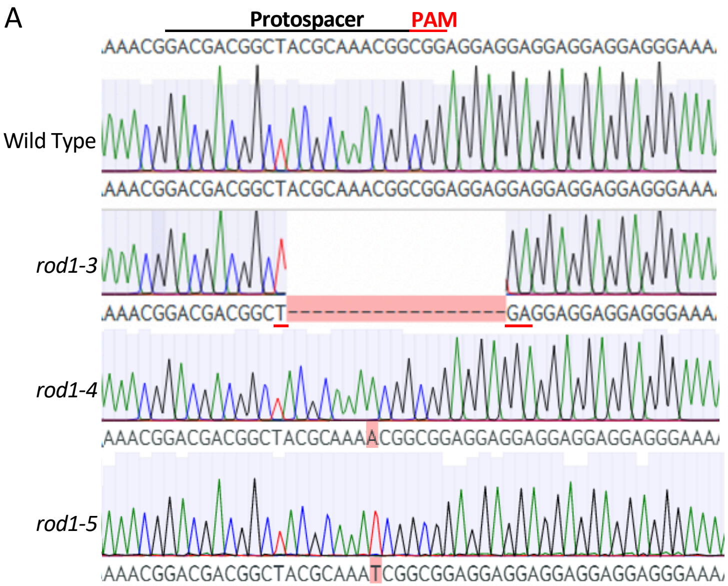

B

Wild-Type ROD1 polypeptide sequence

```
MSTKTVVPLRRRSKPLNGNHTNGVAID
GSLDDDHNRRIGSVNSQMDNIAKKTDD
GYANGGGGGGGGKSKASFMTWTARDVV
YVARYHWIPCLFAVGVLFFTGVEYTLQ
MIPARSEPFDIGFVATRSLNRVLANSP
DLNTVLAALNTVFVGMQTTYIVWTWLM
EGRPRATISACFMFTCRGILGYSTQLP
LPQDFLGSGVDFPVGNVSFFLFYSGHV
AGSMIASLDMRRMQRMRLAMLFDILNV
LQSIRLLGTRGHYTIDLAVGVGAGILF
DSFAGKYEEMISKRHNLVNGFGLISKD
SLVN-
```

C

rod1-3 predicted polypeptide sequence

```
STKTVVPLRRRSKPLNGNHTNGVAIDGS
LDDDHNRRIGSVNSQMDNIAKKTDDG-
```

D

rod1-4 predicted polypeptide sequence

```
MSTKTVVPLRRRSKPLNGNHTNGVAID
GSLDDDHNRRIGSVNSQMDNIAKKTDD
GYAKRRRRRRRREKQGVVYDVDGA-
```

E

rod1-5 predicted polypeptide sequence

```
MSTKTVVPLRRRSKPLNGNHTNGVAIDGSLDD
DHNRRIGSVNSQMDNIAKKTDDGYANRRRRRR
RREKQGVVYDVDGA-
```

Figure S4. **C**haracterization of the CRISPR-induced *Thlaspi arvense rod1* mutants' sequences. (**A**) *Tarod1* DNA sequence chromatograms showing the nature of each mutation. *TaROD1* coding sequences homozygous for wild type (top sequence); 18 bp deletion (*rod1-3*); +A insertion (*rod1-4*); and +T insertion (*rod1-5*). All three mutations are located as expected at the CRISPR/*Sp*Cas9 guide RNA binding site ("Protospacer" location delineated with a black line). (**B, C, D, E**) Predicted ROD1 polypeptide sequence in (**B**) wild-type pennycress versus the **(C)** *rod1-3* **(D)** *rod1-4*, and **(E)** *rod1-5* mutants. Note the predicted polypeptides encoded by *rod1-4* and *rod1-5* are truncated due to frameshifts in the open reading frame (ORF), whereas the 18 bp deletion in *rod1-4* introduces a premature stop codon (underlined in red).

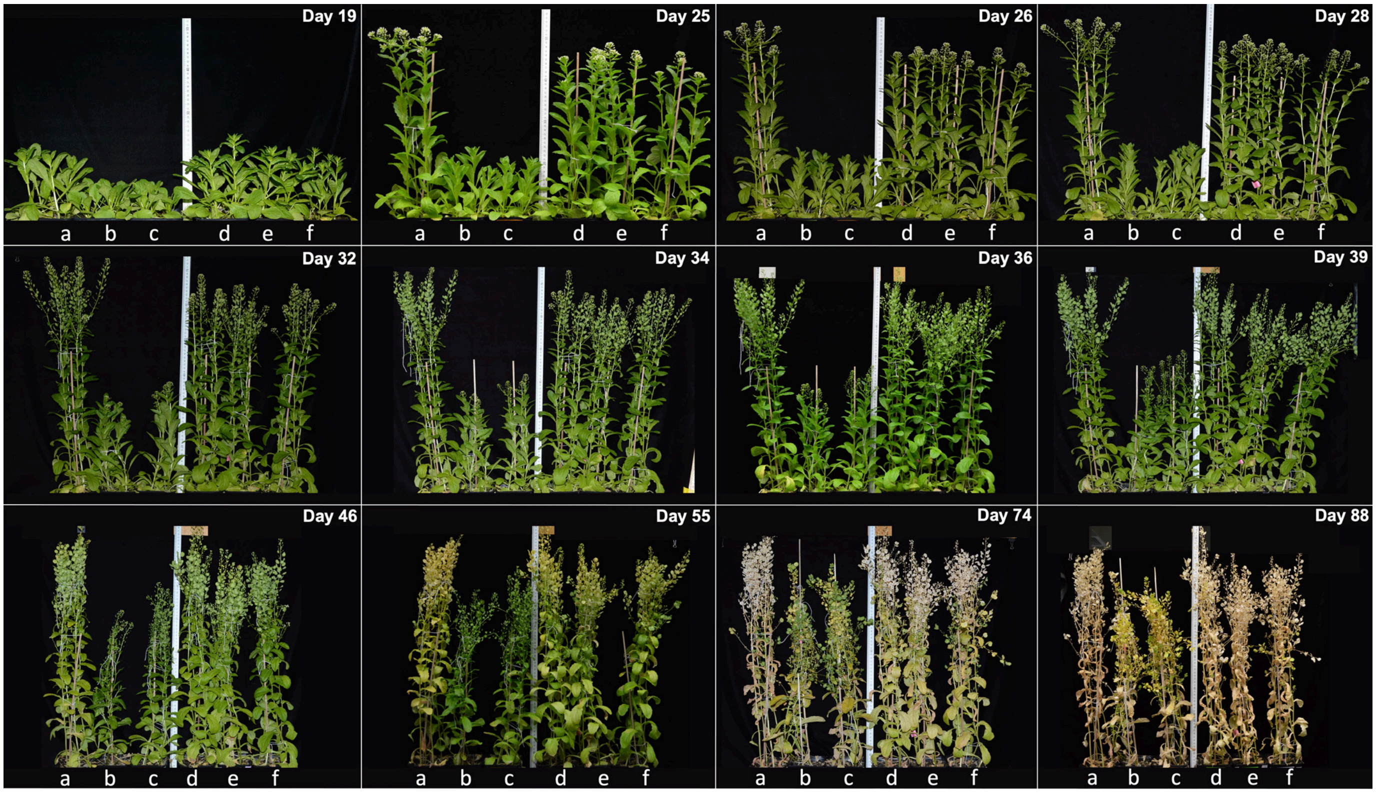

Figure S5. Growth time course of different lipid mutants and wild-type plants grown at the same time and conditions from Day 19 until Day 88 of their plant life cycles. Shown are four plants for each genotype growing in four-inch pots. (a) *rod1 fae1*, (b) *fad2 fae1*, (c) *fad2*, (d) Wild-type Spring 32-10, (e) *fae1*, and (f) *rod1*.

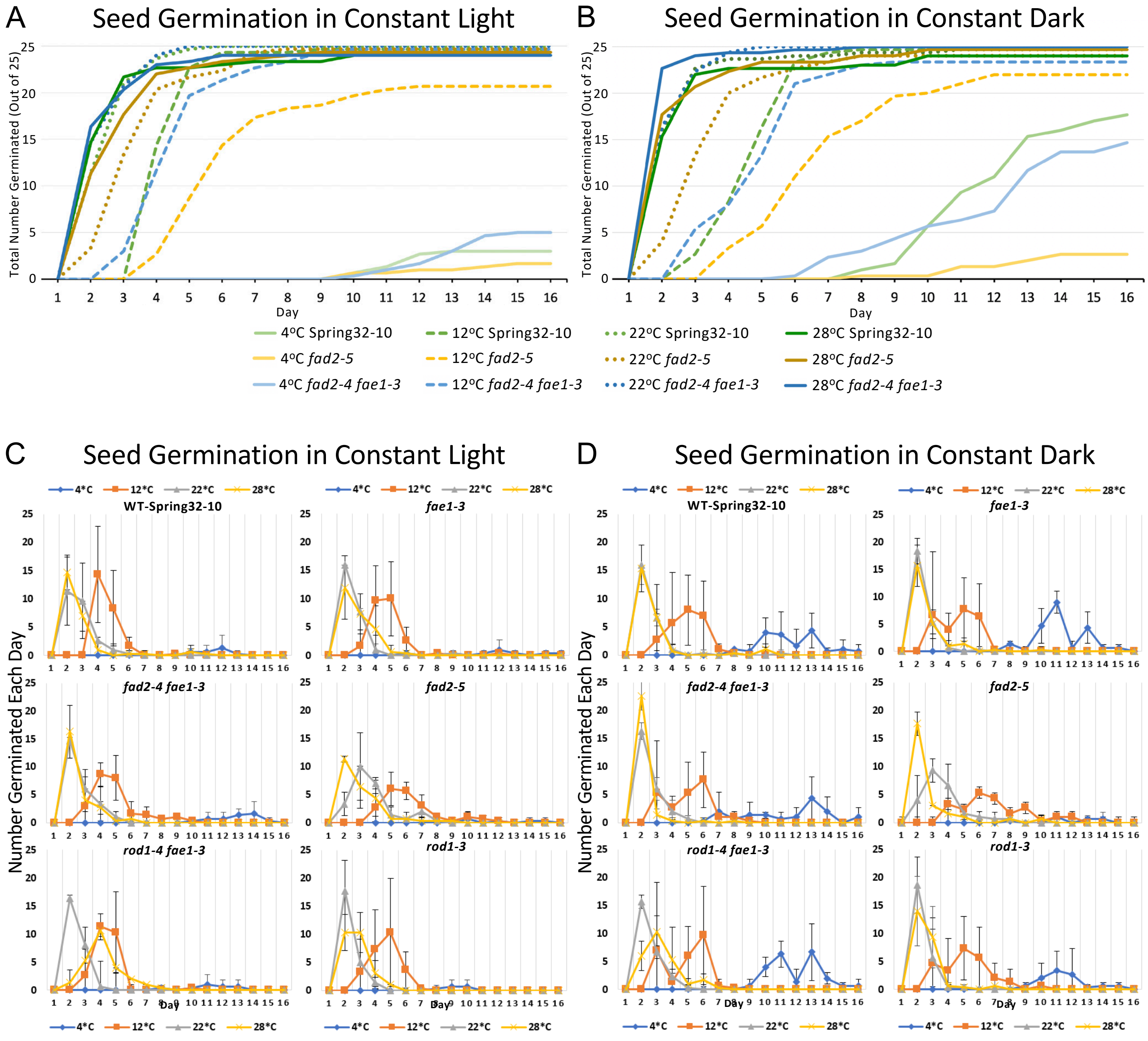

Figure S6A-D. Average amounts of seed germination over a 16-day period under different temperatures and light regimes. Sets of 75 seeds for each genotype were plated onto three agar growth media plates (25 seeds per plate; n = 3) and incubated at 4 ºC, 12 ºC, 22 ºC, or 28 ºC either in constant florescent light (**A, C**) or constant darkness (**B, D**). Seed germination in (**A**) and (**B**) are graphed as cumulative, whereas as (**C**) and (**D**) are graphed as the number that germinated each day. Values and significant differences can be found in Tables S2 and S3. Error bars in (**C**) and (**D**) are standard deviations.

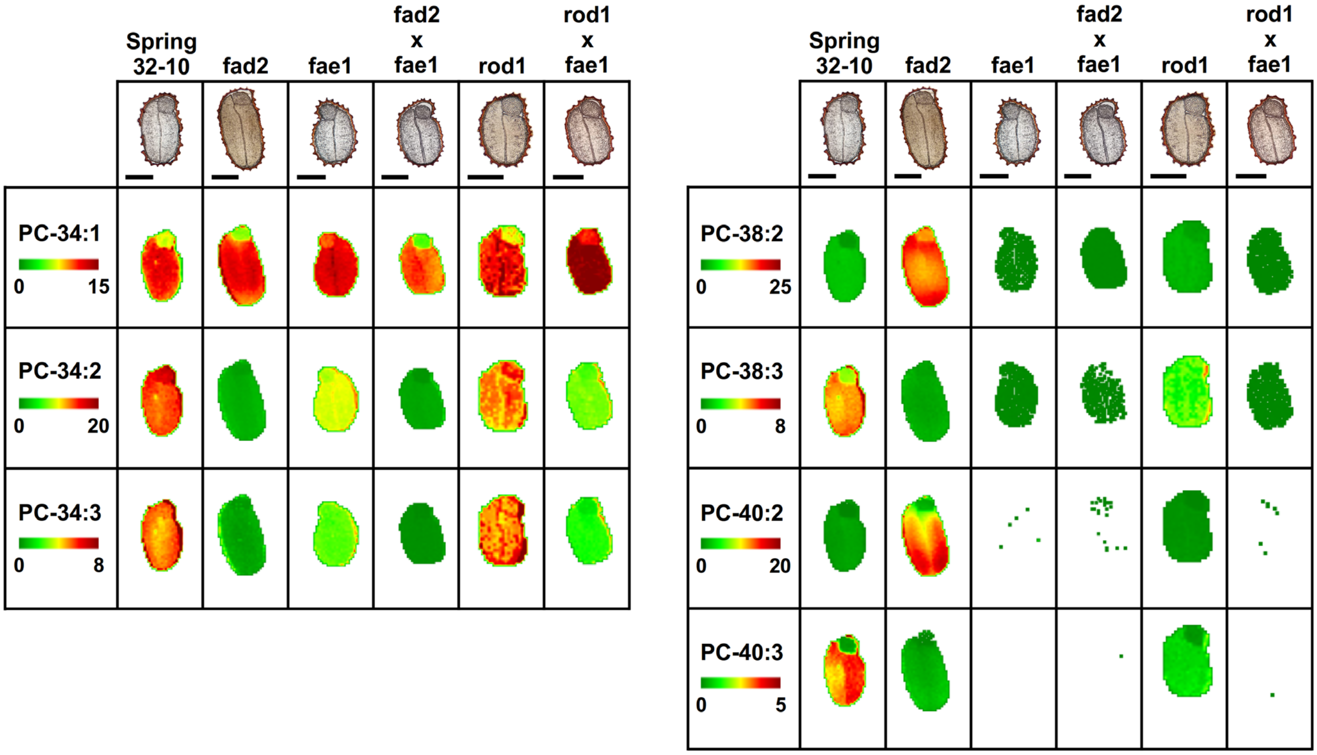

Figure S7. MS imaging of other PC molecular species detected in wild-type Spring 32-10, the various mutants and mutant combinations.

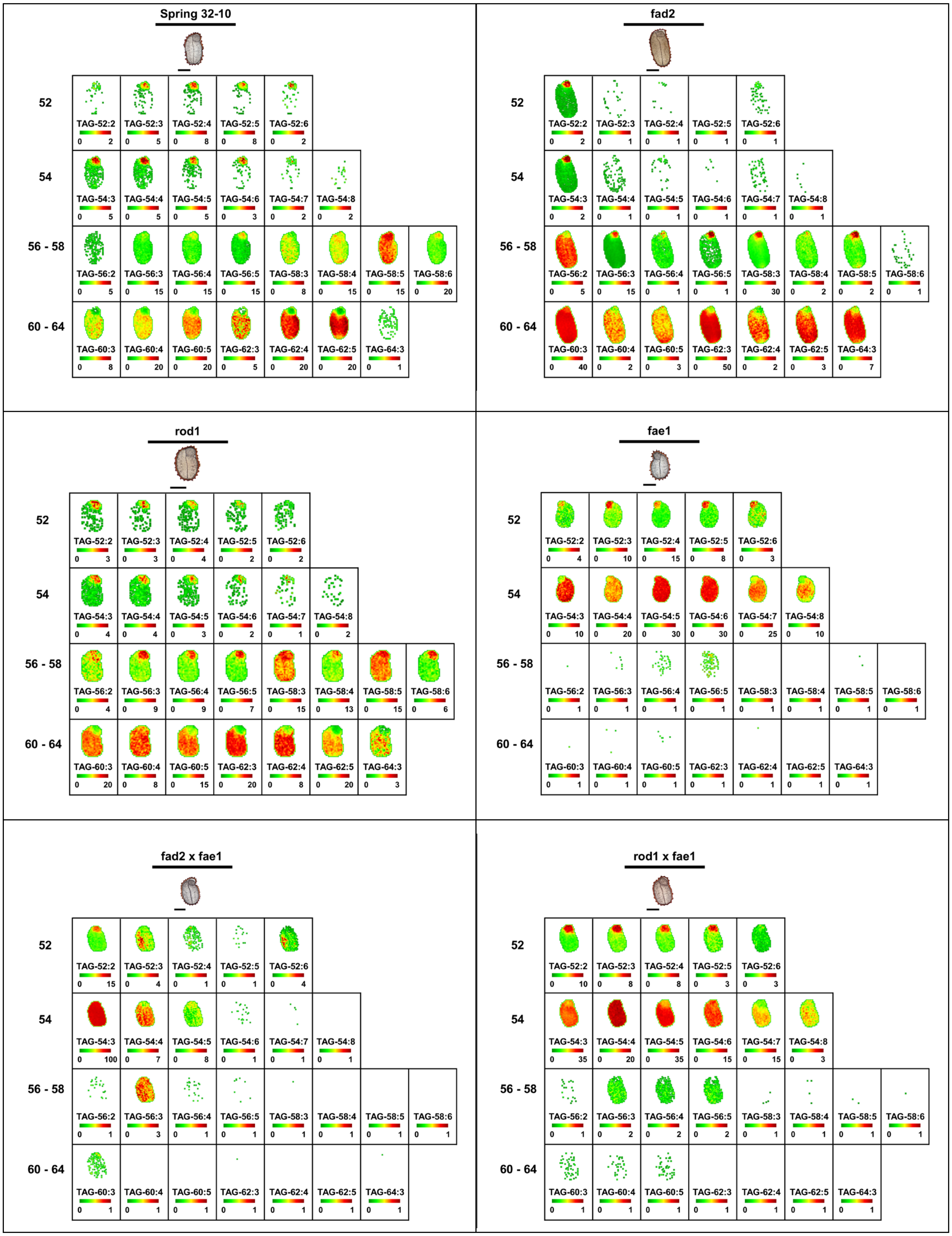

Figure S8. MS imaging of each TAG molecular species detected for wild type and mutant seeds.
